# Nur77 Is Associated With Polyfunctional Properties in Virus‐Specific Human CD8^+^ T Cells

**DOI:** 10.1002/eji.70202

**Published:** 2026-05-12

**Authors:** Fabiola Martel, Daniel Rincón, Caroline Passaes, Leonardo Arévalo, Marcela Torres, Natalia Ramirez, Carlos F. Narváez, Jessica F. Toro, Otto Sussmann, Manuel A. Franco, José Mateus, John Sidney, Alessandro Sette, Asier Sáez‐Cirión, Federico Perdomo‐Celis

**Affiliations:** ^1^ Instituto de Genética Humana Facultad de Medicina Pontificia Universidad Javeriana Bogotá Colombia; ^2^ CellRep Corporation Dover Delaware USA; ^3^ Institut Pasteur Université Paris Cité Viral Reservoirs and Immune Control Unit Paris France; ^4^ Institut Pasteur Université Paris Cité HIV Inflammation and Persistence Unit Paris France; ^5^ Infectoclinicos SAS Bogota Colombia; ^6^ División de Inmunología Programa de Medicina Facultad de Ciencias de La Salud Universidad Surcolombiana Neiva Huila Colombia; ^7^ Servicio de Pediatría Clínica Medilaser Neiva Colombia; ^8^ Vividion Therapeutics San Diego California USA; ^9^ Center for Infectious Disease and Vaccine Research La Jolla Institute for Immunology La Jolla California USA

**Keywords:** CD8^+^ T cells, human, memory, Nur77, polyfunctionality, proliferation, stem‐like

## Abstract

Human CD8^+^ T cells undergo significant metabolic and transcriptional shifts during activation and differentiation. The orphan nuclear receptor Nur77 plays a role in modulating these processes and has been linked to T cell dysfunction. However, few studies have addressed its role in the memory potential and functionality of human CD8^+^ T cells. Here, we evaluated the expression of Nur77 in human CD8^+^ T cells, focusing on its relationship with their differentiation profile and functionality. Our findings indicate that Nur77 is associated with an early‐differentiated, T cell factor 1^+^ (TCF‐1^+^) memory‐like phenotype in both total and virus‐specific human CD8^+^ T cells across contexts of acute resolved or chronic viral infection and vaccination. Nur77 expression was associated with cytokine polyfunctionality and increased proliferative capacity in long‐lived antigen‐responsive cells. Moreover, the modulation of Nur77 activity in vitro enhanced the functionality of chronic human immunodeficiency virus (HIV) and hepatitis B virus (HBV)‐specific CD8^+^ T cells. These results suggest that Nur77 is associated with polyfunctional properties in virus‐specific CD8^+^ T cell responses in humans. In addition, this study provides insights into novel strategies for enhancing CD8^+^ T cell functionality in settings of chronic antigen stimulation.

## Graphical abstract

Nur77 is associated with a TCF‐1^+^ memory‐like, polyfunctional profile in virus‐specific human CD8^+^ T cells across acute‐resolved, vaccinated, and chronic viral infections, and its pharmacologic modulation in vitro enhances the functionality of dysfunctional HIV‐ and HBV‐specific CD8^+^ T cells in chronic co‐infections.

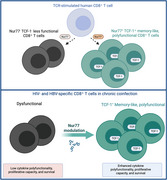

## 1 Introduction

Multiple transcriptional and metabolic factors regulate CD8^+^ T cell differentiation and functionality. For instance, anabolic metabolism regulators, such as the mTORC signaling pathway and glucose metabolism, regulate effector versus memory‐like responses [1]. Nur77 (*NR4A1*) is a member of the NR4A family of orphan nuclear receptors that function as transcription factors capable of inducing or suppressing gene transcription associated with T cell metabolism and effector differentiation [2]. Classically, Nur77 has been employed as a marker of T cell receptor (TCR)‐mediated activation in T cells [3]. Moreover, Nur77 has been identified as a key modulator of T cell activation and effector differentiation in mouse models. As such, Nur77 is activated following TCR stimulation and regulates the metabolic shift during T cell activation by regulating the expression of genes involved in electron transport and glucose metabolism [4, 5]. In addition, recent studies have shown that NR4A1 members regulate T cell dysfunction in contexts of tolerance and chronic antigen stimulation [6]. Indeed, mouse studies have shown that exhausted CD8^+^ T cells upregulate NR4A1 during chronic viral infection [7, 8], and deletion of NR4A transcription factors in chimeric antigen receptor (CAR)‐T cells or tumor‐infiltrating T cells promotes tumor regression and prolongs survival of tumor‐bearing mice [9, 10]. Thus, these studies suggest a role of the NR4A family in the development of T cell dysfunction or exhaustion, and the inhibition of Nur77 in cancer immunotherapy has emerged as an alternative to improve the effectiveness of T cell–based therapies [11].

In contrast, recent mouse studies have shown that CD62L^hi^ TCF‐1^hi^ (stem‐like) memory CD8^+^ T cells actively regulate their anabolic metabolism to prevent cellular damage, which is associated with higher Nur77 expression compared to more differentiated CD62L^lo^ TCF‐1^lo^ cells [12]. More recently, it was demonstrated that *NR4A1* is expressed by tumor antigen‐specific progenitor‐exhausted CD8^+^ T (Tpex) cells and *NR4A1* overexpression promotes the development of CD8^+^ Tpex cells. Mechanistically, *NR4A1* upregulates a Tpex transcriptional signature and represses terminally exhausted cell (Tex)‐associated genes, helping to sustain the CD8^+^ T cell response in the tumor microenvironment [13].

These studies support the notion that Nur77 is implicated in the regulation of anabolic metabolism and the generation and/or maintenance of memory/Tpex versus effector/exhausted CD8^+^ T cell responses. However, the relationship of Nur77 with the effector‐like versus memory‐like profile and functionality of human CD8^+^ T cells has not been explored. Here, we show that Nur77 expression is associated with an early‐differentiated, memory‐like profile in total and antigen‐specific human CD8^+^ T cells in contexts of acute resolved or chronic viral infection and vaccination. In addition, Nur77 modulation in dysfunctional virus‐specific CD8^+^ T cells from people with chronic infection enhances their functional properties.

## 2 Results

### 2.1 Nur77 Is Enriched in Early‐Differentiated T Cell Factor 1^+^ (TCF‐1^+^) Human CD8^+^ T Cells

The expression of Nur77 on T cells appears to be rapidly induced upon antigen stimulation [14]. However, few studies have characterized the dynamics of Nur77 expression on human CD8^+^ T cells and the relationship with their differentiation profile. We evaluated Nur77 expression in total purified CD8^+^ T cells from healthy donors upon polyclonal stimulation with anti‐CD3/CD28 antibodies for 48 h. We observed that Nur77 expression correlated with increasing concentrations of anti‐CD3/CD28 antibodies, whereas there was no Nur77 expression in nonactivated cells (Figure S1A). In addition, we stimulated total CD8^+^ T cells with anti‐CD3/CD28 antibodies in the presence or absence of hrICAM‐1, which is known to contribute to the immunological synapse and increase TCR‐mediated signaling via lymphocyte function‐associated antigen 1 binding [15]. The stimulation in the presence of hrICAM‐1 resulted in an enhanced expression of Nur77 on total CD8^+^ T cells, and this was particularly evident at lower doses of anti‐CD3/CD28 antibodies (Figure S1B). These data confirm that Nur77 is upregulated in human CD8^+^ T cells upon TCR activation.

**FIGURE 1:**
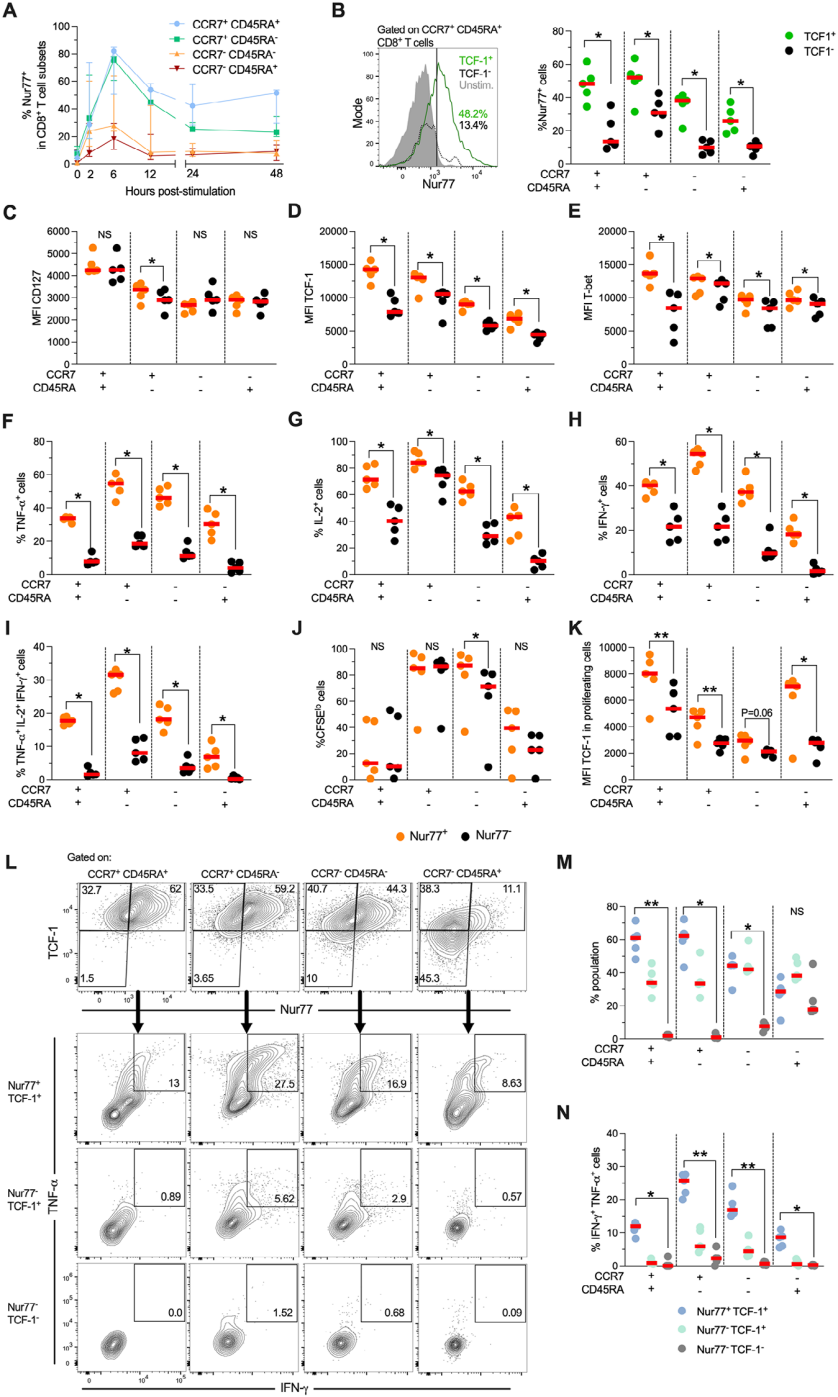
Nur77 is enriched in early‐differentiated human CD8^+^ T cells. (A) Expression of Nur77 in CD8^+^ T cell subsets (according to the expression of CCR7 and CD45RA) upon stimulation with anti‐CD3/CD28 antibodies (1 µg/mL) for up to 48 h (*n* = 3). (B) Left panels: Representative expression of Nur77 in TCF‐1^+^ and TCF‐1^−^ CCR7^+^ CD45RA^+^ CD8^+^ T cells. Right panel: Frequencies of Nur77^+^ cells among TCF‐1^+^ and TCF‐1^−^ CD8^+^ T cell subsets. (C–E) Expression of CD127 (C), TCF‐1 (D), and T‐bet (E) in Nur77^+^ and Nur77^−^ populations among CD8^+^ T cell subsets upon polyclonal stimulation for 48 h. (F–J) Frequency of TNF‐α^+^ (F), IL‐2^+^ (G), IFN‐γ^+^ (H), TNF‐α^+^ IL‐2^+^ IFN‐γ^+^ (I), and CFSE^lo^ (J) cells in Nur77^+^ and Nur77^−^ populations among CD8^+^ T cell subsets upon polyclonal stimulation for 48 h. (K) CD8^+^ T cells were stimulated with anti‐CD3/CD28 antibodies for 96 h. The median fluorescence intensity (MFI) of TCF‐1 among Nur77^+^ and Nur77^−^ CD8^+^ T cell subsets is shown. (L) Representative gating for the analysis of Nur77^+^ TCF‐1^+^, Nur77^−^ TCF‐1^+^, and Nur77^−^ TCF‐1^−^ cells in CD8+ T cell subsets. (M) Frequency of Nur77^+^ TCF‐1^+^, Nur77^−^ TCF‐1^+^, and Nur77^−^ TCF‐1^−^ subsets among CD8^+^ T cell subsets. (N) Frequency of IFN‐γ^+^ TNF‐α^+^ cells among Nur77^+^ TCF‐1^+^, Nur77^−^ TCF‐1^+^, and Nur77^−^ TCF‐1^−^ subsets. In (B–K) and (M–N), the *p* value of Wilcoxon test is shown. Data derived from at least two independent experiments. Symbols represent one individual, and lines indicate the median. TCF‐1^+^, T cell factor 1^+^; NS, not statistically significant. *p < 0.05; **p < 0.01.

We next evaluated the kinetics of expression of Nur77 and the relationship with the differentiation profile of human CD8^+^ T cells (evaluated by CCR7 and CD45RA markers). In keeping with previous studies [4], polyclonal stimulation induced a rapid upregulation of Nur77, peaking at 6 h post‐activation (Figure 1A). Interestingly, there was a graded expression of Nur77 along CD8^+^ T cell subsets, with the highest expression being observed in early‐differentiated CCR7^+^ CD45RA^+^ cells (that contain naïve and stem cell memory cells), followed by CCR7^+^ CD45RA^−^ (central memory) cells (Figure 1A). In contrast, late‐differentiated effector memory (CCR7^−^ CD45RA^−^) and terminal effector memory (CCR7^−^ CD45RA^+^) cells showed a lower expression of Nur77 (Figure 1A). In addition, relative to late‐differentiated cells, CCR7^+^ CD45RA^+^ and CCR7^+^ CD45RA^−^ cells maintained a higher Nur77 expression upon 48 h of stimulation (Figure 1A). This relationship between Nur77 expression and the human CD8^+^ T cell differentiation profile was also observed in the presence or absence of hrICAM‐1 co‐stimulation (Figure S1C). The results indicate that Nur77 expression is increased in early‐differentiated memory‐like CD8^+^ T cell subsets following polyclonal stimulation. It is important to note that Nur77 expression decreased at later time points. However, the pattern of Nur77 expression among CD8^+^ T cell populations was still observable at these later time points. Thus, subsequent experiments were conducted with stimulations lasting from 48 h up to 6 days. This approach allows us to simultaneously assess cytokine production and proliferation in both polyclonal and antigen‐specific CD8^+^ T cells.

In addition, we evaluated if Nur77 was associated with the expression of TCF‐1, a transcription factor that characterizes stem‐like memory and early‐differentiated human CD8^+^ T cells [16]. Upon polyclonal stimulation, we analyzed TCF‐1^+^ and TCF‐1^−^ cells among naïve and memory cells. In all the human CD8^+^ T cell subsets, we observed that TCF‐1^+^ cells had a higher expression of Nur77 relative to TCF‐1^−^ cells (Figure 1B). These results suggest that the expression of Nur77 is associated with a TCF‐1^+^ early‐differentiated phenotype in human CD8^+^ T cells.

### 2.2 Polyclonal Nur77^+^ CD8^+^ T Cells Exhibit Higher Polyfunctionality and Maintain TCF‐1 Expression Upon Proliferation

Nur77 has been associated with multiple cellular processes, including cell metabolism, proliferation, differentiation, and apoptosis [11]. Here, we sought to evaluate the association between Nur77 expression and the survival, effector function, and proliferative capacity of human CD8^+^ T cells. Upon polyclonal stimulation, we observed that purified CD8^+^ T cells expressing Nur77 exhibited an increased survival capacity compared to their Nur77^−^ counterpart (Figure S1D). Although no major differences were observed in the expression of CD127 between Nur77^+^ and Nur77^−^ cells (Figure 1C), we observed that Nur77^+^ cells had higher expression of TCF‐1 (Figure 1D) and T‐bet (Figure 1E), the latter known to regulate multiple effector functions in human CD8^+^ T cells [17]. The expression of Nur77 was also associated with enhanced production of TNF‐α, IL‐2, and IFN‐γ, both individually and simultaneously (Figure 1F–I and Figure S1E,F). In terms of proliferation, overall, we observed comparable frequencies of CFSE^lo^ cells between Nur77^+^ and Nur77^−^ cells (Figure 1J and Figure S1G), although the Nur77^+^ subset among CCR7^−^ CD45RA^−^ cells exhibited a higher proportion of proliferating cells than the Nur77^−^ counterpart (Figure 1J). Notably, despite proliferation, Nur77^+^ cells consistently maintained a higher TCF‐1 expression relative to Nur77^−^ cells among all CD8^+^ T cell subsets (Figure 1K). Collectively, these results suggest that Nur77 expression in human CD8^+^ T cells is associated with enhanced polyfunctionality and sustained TCF‐1 expression following proliferation.

We also explored the relationship between Nur77 and TCF‐1 expression in CD8^+^ T cell subsets. As shown in Figure 1L, upon polyclonal stimulation, we identified the Nur77^+^ TCF‐1^+^, Nur77^−^ TCF‐1^+^, and Nur77^−^ TCF‐1^−^ populations. We observed a graded proportion of these subsets, with CCR7^+^ CD45RA^+^ and CCR7^+^ CD45RA^−^ cells being enriched in Nur77^+^ TCF‐1^+^ cells (Figure 1L,M). In contrast, late‐differentiated CCR7^−^ CD45RA^+^ cells had a higher proportion of Nur77^−^ TCF‐1^−^ cells (Figure 1M). In terms of their functional profile, Nur77^+^ TCF‐1^+^ had higher polyfunctional capacity than Nur77^−^ TCF‐1^+^ and Nur77^−^ TCF‐1^−^ populations, and this pattern was observed in all memory CD8^+^ T cell subsets (Figure 1N). These data indicate that Nur77 and TCF‐1 co‐expression is associated with a less differentiated profile and higher polyfunctionality in CD8^+^ T cells upon polyclonal stimulation.

### 2.3 Nur77 Characterizes Polyfunctional Memory‐Like Virus‐Specific CD8^+^ T Cells Upon Acute‐Resolved Natural Infection or Vaccination

**FIGURE 2:**
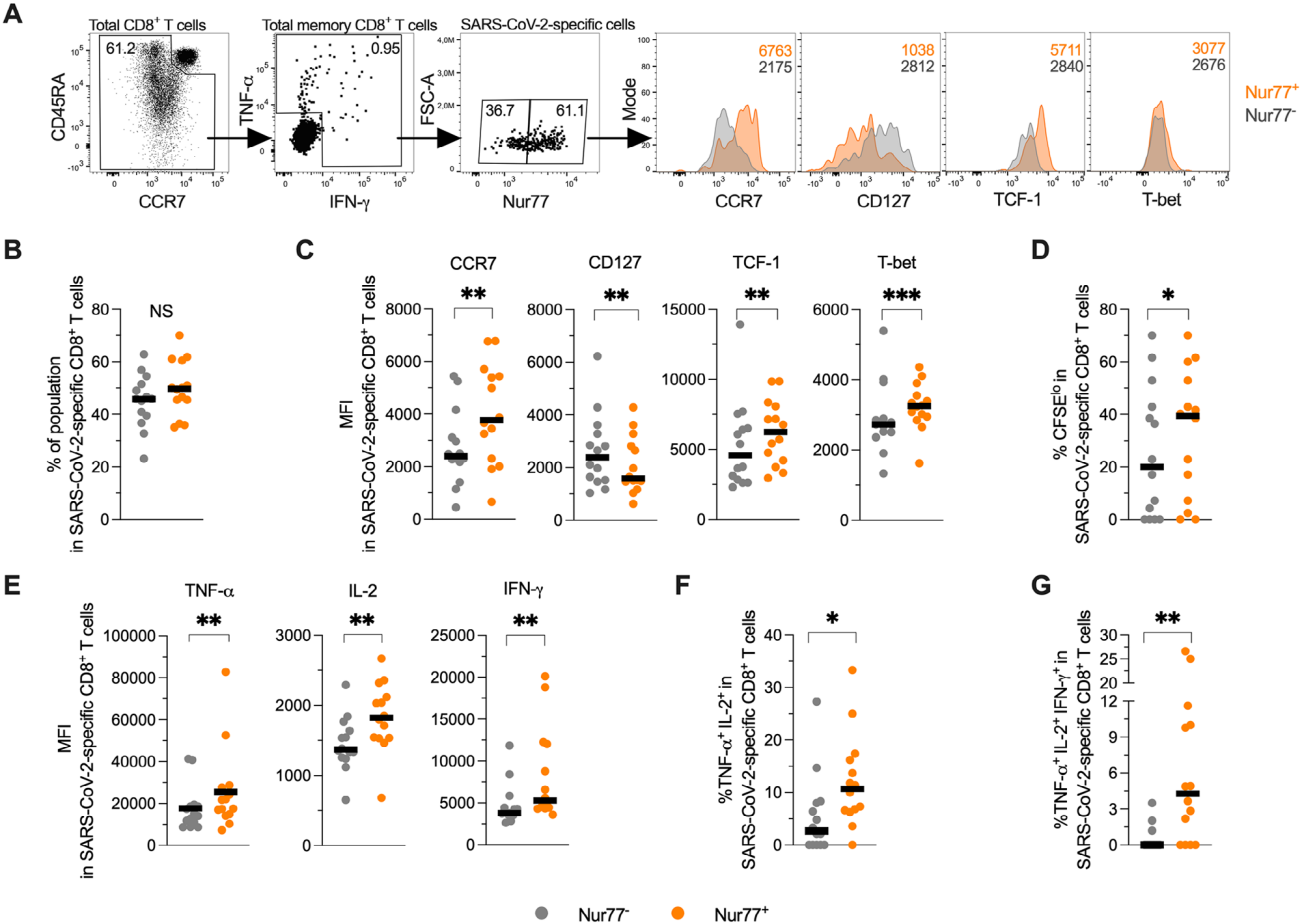
Nur77 is associated with polyfunctional SARS‐CoV‐2‐specific CD8^+^ T cell responses. Peripheral blood mononuclear cells from human donors with previous exposure to SARS‐CoV‐2 were stimulated for 6 days with a peptide megapool derived from the proteome of the SARS‐CoV‐2 Wuhan ancestral strain to assess the naturally induced CD8^+^ T cell response. (A) Representative gating strategy and expression of selected markers in Nur77^+^ and Nur77^−^ SARS‐CoV‐2‐specific CD8^+^ T cells. (B) Frequencies of Nur77^+^ and Nur77^−^ cells among SARS‐CoV‐2‐specific CD8^+^ T cells. (C) Expression of CCR7, CD127, TCF‐1, and T‐bet in Nur77^+^ and Nur77^−^ SARS‐CoV‐2‐specific CD8^+^ T cells. (D) Frequency of CFSE^lo^ cells in Nur77^+^ and Nur77^−^ SARS‐CoV‐2‐specific CD8^+^ T cells. (E) Expression of TNF‐α, IL‐2, and IFN‐γ in Nur77^+^ and Nur77^−^ SARS‐CoV‐2‐specific CD8^+^ T cells. (F–G) Frequency of TNF‐α^+^ IL‐2^+^ (F) and TNF‐α^+^ IL‐2^+^ IFN‐γ^+^ (G) cells in Nur77^+^ and Nur77^−^ SARS‐CoV‐2‐specific CD8^+^ T cells. In (B–G), the *p* value of Wilcoxon test is shown. Data derived from four independent experiments. Symbols represent one individual, and lines indicate the median. NS, not statistically significant; SARS‐CoV‐2, severe acute respiratory syndrome coronavirus 2. *p < 0.05; **p < 0.01; ***p < 0.001.

Having characterized Nur77‐expressing polyclonal CD8^+^ T cells, we evaluated the association between Nur77 expression and the differentiation and functional profile of antigen‐specific memory cells. For this, we analyzed samples from human donors with a history of natural infection of severe acute respiratory syndrome coronavirus 2 (SARS‐CoV‐2; Figure S2A,B). CD8^+^ T cells were stimulated with a peptide megapool derived from the proteome of the SARS‐CoV‐2 Wuhan ancestral strain (excluding the S protein) to assess the naturally induced CD8^+^ T cell response. To evaluate the memory potential of virus‐specific CD8^+^ T cells, in our study, we sequentially stimulated these cells in a 6‐day interval experiment and evaluated the proliferative capacity along with cytokine production of responding cells, as previously reported [18, 19]. SARS‐CoV‐2‐specific CD8^+^ T cells were defined as IFN‐γ^+^ and/or TNF‐α^+^ memory CD8^+^ T cells (Figure 2A and Figure S2A), and ∼20% of them exhibited a CCR7^+^ CD45RA^−^ central memory phenotype (Figure S2B). No significant differences were observed in the frequencies of Nur77^+^ versus Nur77^−^ cells among SARS‐CoV‐2 specific CD8^+^ T cells (Figure 2B). However, relative to the Nur77^−^ subset, Nur77^+^ cells exhibited higher expression of TCF‐1, and T‐bet, along with lower CD127 (Figure 2C). Additionally, Nur77^+^ human SARS‐CoV‐2‐specific CD8^+^ T cells showed enhanced proliferative capacity (Figure 2D) and higher cytokine polyfunctionality compared to their Nur77^−^ counterpart (Figure 2F).

We next evaluated if Nur77 is also associated with a memory‐like profile in virus‐specific CD8^+^ T cells induced by vaccination. Upon HBsAg peptide stimulation, we analyzed the hepatitis B virus (HBV)‐specific CD8^+^ T cell response in individuals who received the recombinant HBV vaccine (Figures S2A and S3A). Around 40% of HBV‐specific CD8^+^ T cells exhibited a CCR7^+^ CD45RA^−^ central memory phenotype (Figure S2C). Similar to our findings with SARS‐CoV‐2‐specific CD8^+^ T cells, there were no differences in the proportion of Nur77^+^ and Nur77^−^ subsets within HBV‐specific CD8^+^ T cells (Figure S3B), but Nur77^+^ cells exhibited higher expression of TCF‐1, T‐bet, and lower CD127, relative to Nur77^−^ cells (Figure S3C). Moreover, Nur77^+^ HBV‐specific CD8^+^ T cells showed higher proliferation and a trend for higher polyfunctionality (Figure S3E and S3F). Thus, upon natural infection and vaccination, respectively, Nur77 characterizes SARS‐CoV‐2‐ and HBV‐specific CD8^+^ T cells with memory‐like and polyfunctional properties.

We asked if the relationship between Nur77 and the human CD8^+^ T cell functional profile that we observed on SARS‐CoV‐2‐specific memory cells was also present during an acute viral infection. To solve this question, we analyzed the dengue virus (DENV)‐specific CD8^+^ T cells from pediatric donors during acute infection, upon stimulation with a DENV CD8 peptide megapool set for 6 days (Figures S2A and S4A). Of note, similar to previous studies [20], acute infection DENV‐specific CD8^+^ T cells were highly enriched in a CCR7^−^ CD45RA^−^ effector phenotype (Figure S2D). Upon peptide stimulation, the DENV‐specific CD8^+^ T cell response was dominated by Nur77^−^ CD8^+^ T cells (Figure S4B). On the basis of the expression of Nur77, DENV‐specific CD8^+^ T cells did not show differences in the expression of CCR7, TCF‐1, and T‐bet, although Nur77^+^ cells had lower levels of CD127 (Figure S4C). In addition, overall, we did not observe differences in proliferation and cytokine production between Nur77^+^ and Nur77^−^ cells (Figure S4E and S4F). These results indicate that contrary to virus‐specific memory cells, the expression of Nur77 does not distinguish subsets of virus‐specific effector CD8^+^ T cells.

### 2.4 Nur77 Expression Distinguishes Polyfunctional Virus‐Specific CD8^+^ T Cells During Chronic Infection

We evaluated if Nur77 is also associated with the functionality of human virus‐specific CD8^+^ T cells during chronic infection. To this end, we included individuals with chronic coinfection with the human immunodeficiency virus Type 1 (HIV‐1) and HBV who were under antiretroviral therapy and exhibited undetectable viral load for both viruses. We performed peptide stimulation for 6 days with megapools derived from the whole HIV or HBV proteome and analyzed the virus‐specific CD8^+^ T cell response (Figure S2A). Most of the HIV and HBV‐specific response was enriched in CCR7^−^ CD45RA^−^ cells (Figure S2E,F).

**FIGURE 3:**
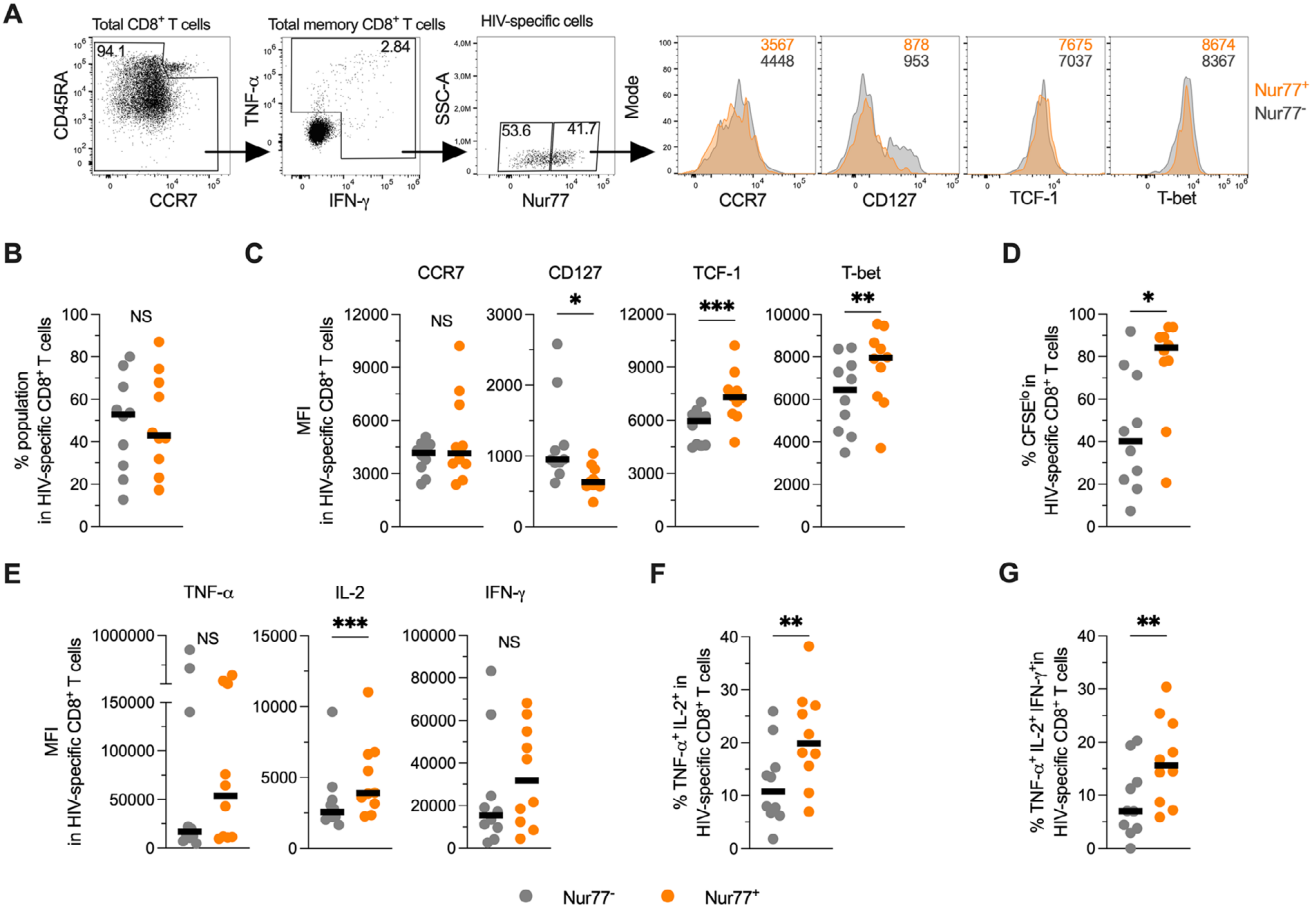
Nur77 is associated with polyfunctional HIV‐specific CD8^+^ T cell responses. Peripheral blood mononuclear cells from people with HIV/HBV coinfection were stimulated for 6 days with a peptide megapool derived from the whole HIV proteome. (A) Representative gating strategy and expression of selected markers in Nur77^+^ and Nur77^−^ HIV‐specific CD8^+^ T cells. (B) Frequencies of Nur77^+^ and Nur77^−^ cells among HIV‐specific CD8^+^ T cells. (C) Expression of CCR7, CD127, TCF‐1, and T‐bet in Nur77^+^ and Nur77^−^ HIV‐specific CD8^+^ T cells. (D) Frequency of CFSE^lo^ cells in Nur77^+^ and Nur77^−^ HIV‐specific CD8^+^ T cells. (E) Expression of TNF‐α, IL‐2, and IFN‐γ in Nur77^+^ and Nur77^−^ HIV‐specific CD8^+^ T cells. (F–G) Frequency of TNF‐α^+^ IL‐2^+^ (F), and TNF‐α^+^ IL‐2^+^ IFN‐γ^+^ (G) cells in Nur77^+^ and Nur77^−^ HIV‐specific CD8^+^ T cells. In (B–G), the *p* value of Wilcoxon test is shown. Data derived from four independent experiments. Symbols represent one individual, and lines indicate the median. HIV, human immunodeficiency virus; NS, not statistically significant. *p < 0.05; **p < 0.01; ***p < 0.001.

No significant differences were observed in the proportion of Nur77^+^ versus Nur77^−^ cells within the human HIV‐specific CD8^+^ T cells (Figure 3A,B). In contrast, chronic HBV‐specific CD8^+^ T cells were enriched in Nur77^−^ cells (Figure S5A,B). Relative to Nur77^−^ cells, Nur77^+^ HIV‐specific CD8^+^ T cells had a higher expression of TCF‐1 and T‐bet, and lower CD127, whereas no differences were observed for CCR7 (Figure 3C). Similarly, Nur77^+^ HBV‐specific CD8^+^ T cells had higher expression of TCF‐1 and lower CD127 in comparison with Nur77^−^ cells (Figure S5C). In addition, Nur77^+^ HIV‐ and HBV‐specific CD8^+^ T cells had a higher proportion of proliferating cells than Nur77^−^ cells (Figure 3D and Figure S5D). In the case of HIV‐specific cells, higher polyfunctionality was observed in Nur77^+^ cells relative to the Nur77^−^ counterpart, whereas a similar trend was observed for HBV‐specific cells (Figure 3F and Figure S5F). Thus, Nur77 characterizes HIV‐ and HBV‐specific CD8^+^ T cells with polyfunctional properties during chronic infection.

Moreover, we directly compared the percentage of Nur77^+^ cells and the expression of TCF‐1 in Nur77‐expressing virus‐specific CD8^+^ T cells upon acute resolved infection (SARS‐CoV‐2‐specific), vaccination (HBsAg‐specific), acute infection (DENV‐specific), and chronic infection (HIV‐ and HBV‐specific). We observed a higher proportion of Nur77^+^ cells in SARS‐CoV‐2‐ and vaccine HBV‐specific cells relative to chronic HBV‐specific cells (Figure S6A). In addition, Nur77^+^ vaccine HBV‐specific cells exhibited a higher expression of TCF‐1 in comparison with SARS‐CoV‐2‐specific, DENV‐specific cells, and chronic HBV‐specific cells (Figure S6B). We also observed a positive correlation between the proportion of Nur77^+^ cells and the frequency of CCR7^+^ CD45RA^−^ central memory cells among virus‐specific CD8^+^ T cells (Figure S6C). These results further suggest that Nur77 is associated with a TCF‐1^+^ long‐lived memory profile during human viral infections.

### 2.5 Nur77 Upregulation in CD8^+^ T Cells Reprogrammed Towards a Memory‐Like Profile

**FIGURE 4:**
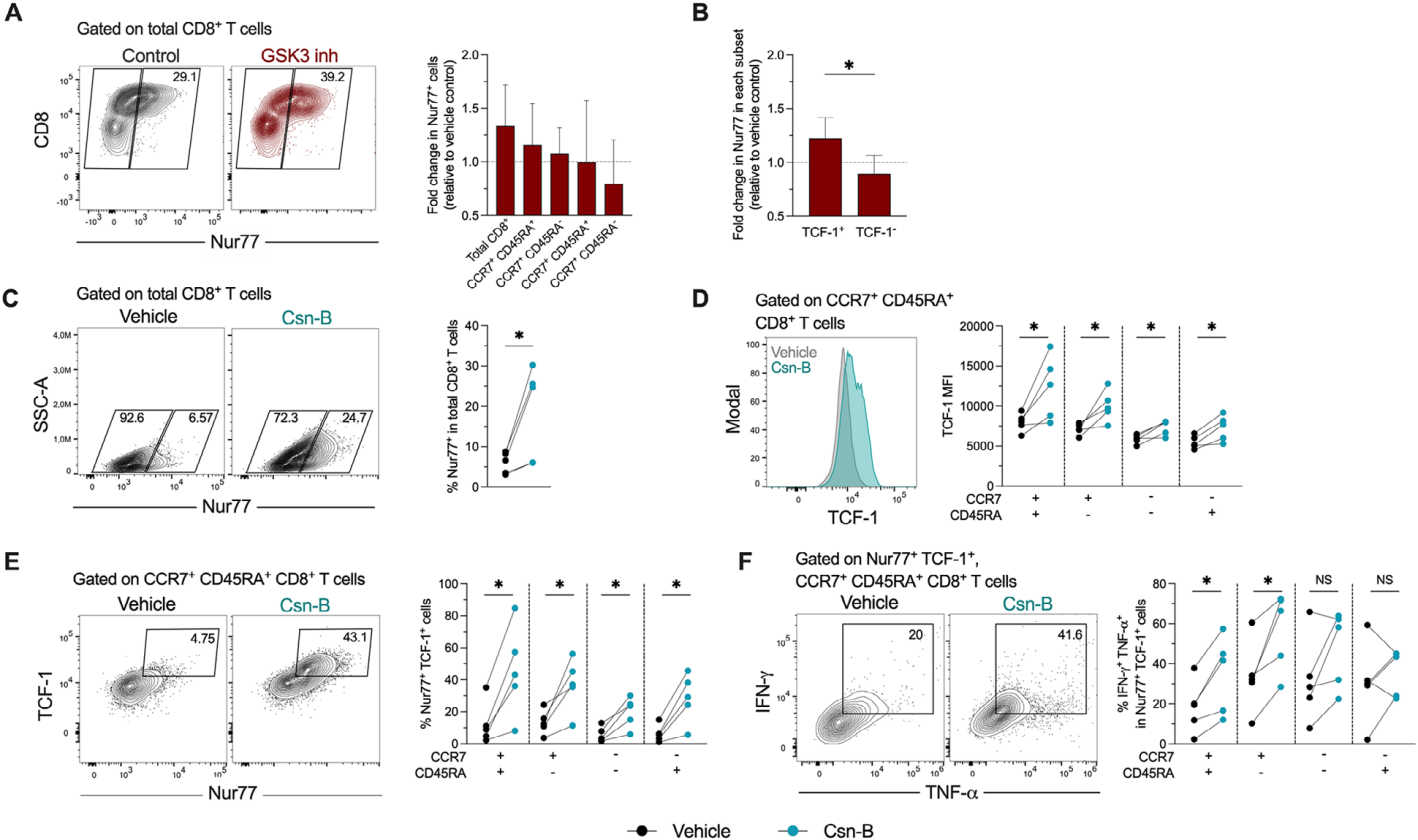
Nur77 modulation promotes Nur77^+^ TCF‐1^+^ polyfunctional human CD8^+^ T cells. Purified CD8^+^ T cells from healthy donors were treated with vehicle control or reprogrammed with a GSK3 inhibitor, followed by stimulation with anti‐CD3/CD28 antibodies and hrICAM‐1, for 48 h. (A) Left: representative expression of Nur77 in reprogrammed and non‐reprogrammed CD8^+^ T cells, upon polyclonal stimulation. Right: fold change in the frequency of Nur77^+^ cells in reprogrammed CD8^+^ T cells, relative to non‐reprogrammed cells. (B) Fold change in Nur77 expression in TCF‐1^+^ or TCF‐1^−^ reprogrammed CD8^+^ T cells, relative to non‐reprogrammed cells. In (A) and (B), the median and interquartile ranges are shown (*n* = 8). (C–F) Purified CD8^+^ T cells from healthy donors were polyclonally stimulated with anti‐CD3/CD28 antibodies in the presence or absence of cytosporone B (Csn‐B), for 48 h. (C) Left: representative expression of Nur77 upon stimulation in Csn‐B‐treated and untreated cells. Right: frequency of Nur77^+^ CD8^+^ T cells upon the indicated treatments. (D) Expression of TCF‐1 upon polyclonal stimulation in Csn‐B‐treated and untreated CD8^+^ T cell subsets. (E–F) Frequencies of Nur77^+^ TCF‐1^+^ cells (E), and IFN‐γ^+^ TNF‐α^+^ cells among Nur77^+^ TCF‐1^+^ CD8^+^ T cell subsets (F), in Csn‐B‐treated and untreated cells. In (B–F), the *p* value of Wilcoxon test is shown. Data derived from at least two independent experiments. Symbols represent one individual, and lines indicate the median. TCF‐1^+^, T cell factor 1^+^; NS, not statistically significant. *p < 0.05.

We previously demonstrated that GSK3 inhibition in vitro of human CD8^+^ T cells induces reprogramming towards polyfunctional memory‐like cells [18]. To further elucidate the role of Nur77 on human CD8^+^ T cell differentiation, we treated purified total CD8^+^ T cells with a GSK3 inhibitor, followed by polyclonal stimulation with anti‐CD3/CD28 antibodies and hrICAM‐1. In line with our previous study [18], GSK3 inhibition promoted a higher expression of TCF‐1 upon polyclonal stimulation and increased the frequency of polyfunctional IL‐2^+^ TNF‐α^+^ cells (Figure S7A,B). In addition, CD8^+^ T cell reprogramming towards a memory‐like profile was associated with an enhanced expression of Nur77 in CD8^+^ T cells (Figure 4A), with a larger effect on early‐differentiated CCR7^+^ CD45RA^+^ and CCR7^+^ CD45RA^−^ cells (Figure 4A), as well as on TCF‐1^+^ cells (Figure 4B). Thus, in line with our results on antigen‐specific CD8^+^ T cells, the in vitro reprogramming towards polyfunctional memory‐like cells is associated with an enhanced expression of Nur77.

### 2.6 Nur77 Modulation Promotes Polyfunctional TCF‐1‐Expressing Memory‐Like CD8^+^ T Cells

After establishing an association between Nur77 expression and memory‐like traits in virus‐specific human CD8^+^ T cells, we explored the effect of cytosporone B (Csn‐B), a small molecule that has been shown to promote Nur77 transcriptional activity [21], on the functionality of human CD8^+^ T cells. Purified CD8^+^ T cells were polyclonally stimulated in the presence or absence of Csn‐B. As expected, we observed a higher expression of Nur77 in CD8^+^ T cells after Csn‐B treatment (Figure 4C), demonstrating a dose‐dependent effect (Figure S7C). In addition, Csn‐B enhanced the expression of TCF‐1 in all human CD8^+^ T cell subsets (Figure 4D). In line with these data, we observed a significant increase in the frequency of Nur77^+^ TCF‐1^+^ cells in all human CD8^+^ T cell subsets (Figure 4E). In addition, upon treatment with Csn‐B, the population of Nur77^+^ TCF‐1^+^ cells in early‐differentiated CCR7^+^ CD45RA^+^ and CCR7^+^ CD45RA^−^ cells exhibited increased polyfunctionality relative to untreated cells (Figure 4F). This effect was not observed in late‐differentiated cells (Figure 4F). Csn‐B treatment further increased the expression of Nur77^+^ TCF‐1^+^ in early‐differentiated CD8^+^ T cell subsets reprogrammed with a GSK3 inhibitor (relative to non‐reprogrammed cells; Figure S7E), although there was no further effect on the proportion of Nur77^+^ TCF‐1^+^ cells (Figure S7F) and their functionality (Figure S7G).

**FIGURE 5:**
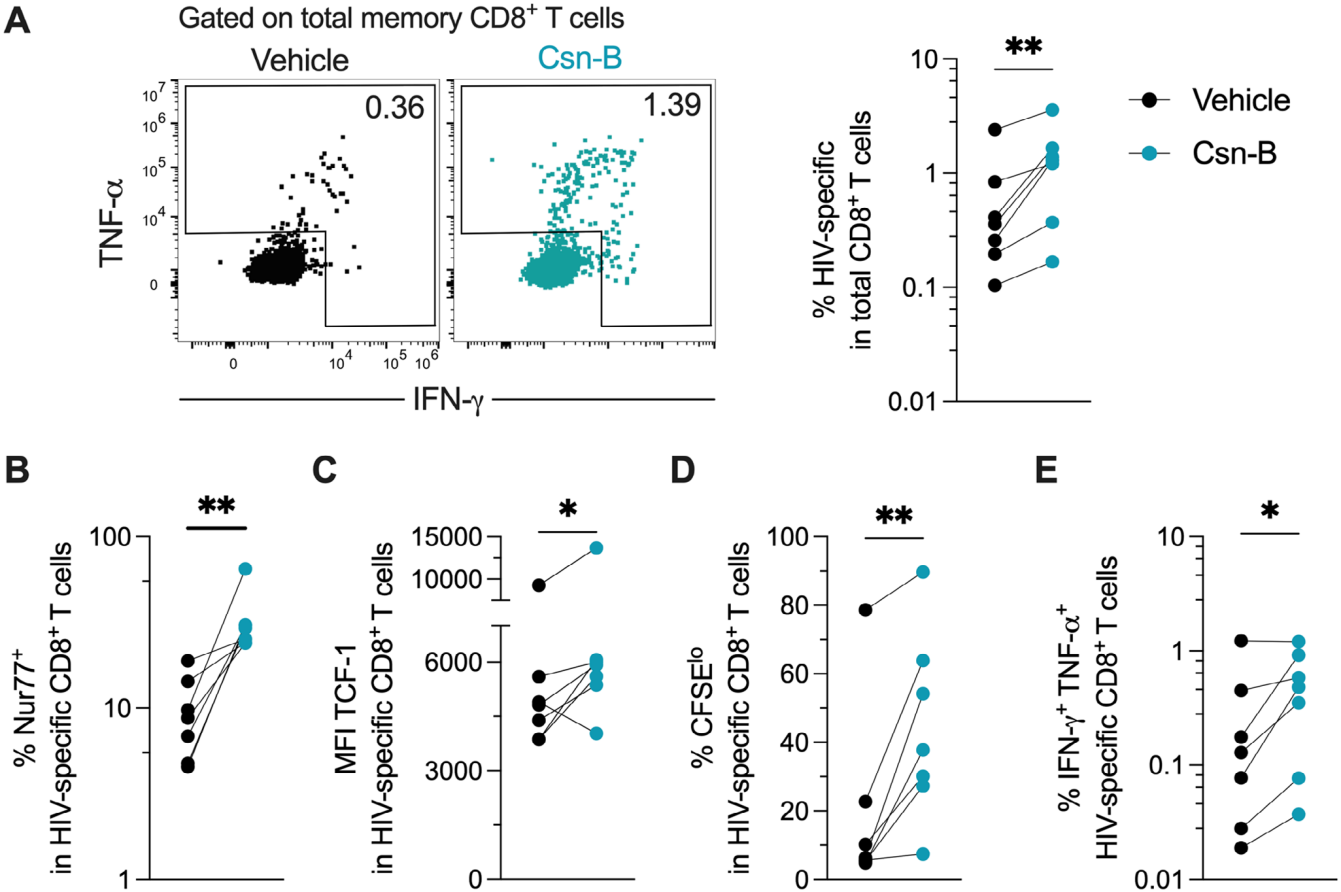
Nur77 modulation enhances the functionality of HIV‐specific CD8^+^ T cells. Peripheral blood mononuclear cells from people with HIV/HBV coinfection were stimulated for 6 days with an HIV peptide megapool in the presence or absence of Csn‐B. (A) Left: representative expression of IFN‐γ and TNF‐α for the analysis of HIV‐specific CD8^+^ T cells. Right: frequency of HIV‐specific CD8^+^ T cells in Csn‐B‐treated and untreated cells. (B) Frequency of Nur77^+^ cells among HIV‐specific CD8^+^ T cells. (C) Expression of TCF‐1 in HIV‐specific CD8^+^ T cells. (D and E) Frequency of CFSE^lo^ (D) and IFN‐γ^+^ TNF‐α^+^ (E) HIV‐specific CD8^+^ T cells. Data derived from two independent experiments. Symbols represent one individual, and lines indicate the median. *p* value of Wilcoxon test. Csn‐B, cytosporone B; HIV, human immunodeficiency virus. *p < 0.05; **p < 0.001.

We next investigated whether Csn‐B treatment enhances the functionality of dysfunctional HIV‐ or HBV‐specific CD8^+^ T cells. CD8^+^ T cells from people with HIV/HBV coinfection were stimulated with the corresponding peptide megapools in the presence or absence of Csn‐B during a 6‐day interval. Compared to vehicle control, Csn‐B treatment significantly increased the total frequency of HIV‐ and HBV‐specific CD8^+^ T cells (Figure 5A and Figure S7H). In addition, Csn‐B increased the proportion of Nur77^+^ cells, the expression of TCF‐1, and the frequency of proliferating cells among HIV‐specific cells (Figure 5B–D). Enhanced polyfunctionality (IFN‐γ^+^ TNF‐α^+^) was also observed in Csn‐B‐treated HIV‐ and HBV‐specific CD8^+^ T cells (Figure 5E and Figure S7I). Furthermore, Csn‐B improved the survival of total peripheral blood mononuclear cells (PBMCs) upon HIV or HBV peptide stimulation (Figure S7J). Both HIV‐ and HBV‐specific CD8^+^ T cells exhibited comparable increases in frequency in response to Csn‐B (Figure S7K). Importantly, the effects of Csn‐B were dependent on TCR‐mediated stimulation by the peptide megapools, as no increase in IFN‐γ^+^ TNF‐α^+^ cells was observed without stimulation (Figure S7K). In addition, the enhancement in IFN‐γ^+^ TNF‐α^+^ and Nur77^+^ TCF‐1^+^ cells, as well as the increased cell survival, was Csn‐B dose‐dependent (Figure S7L). Together, these data indicate that Csn‐B enhances the functional capacity of HIV‐ and HBV‐specific CD8^+^ T cells.

## 3 Discussion

The regulation of CD8^+^ T cell differentiation is crucial for mounting effective immune responses. The balance between effector‐like and memory‐like profiles determines the ability of the immune system to eliminate pathogens while establishing long‐term immunity. In this study, we show that Nur77, previously identified as a T cell dysfunction‐related transcription factor, characterizes polyfunctional memory‐like virus‐specific CD8^+^ T cells in humans. In addition, the treatment with a Nur77 modulator potentiates the effect of in vitro CD8^+^ T cell reprogramming towards TCF‐1‐expressing memory‐like cells, as well as enhances the functionality of dysfunctional virus‐specific CD8^+^ T cells. Overall, our findings suggest that Nur77 is involved in the effector‐like versus memory‐like balance, potentially serving as target for modulating immune responses in various contexts.

Consistent with previous studies [22], Nur77 expression was rapidly upregulated in human CD8^+^ T cells following TCR stimulation, but it was higher in early‐differentiated CCR7^+^ than in late‐differentiated CCR7^−^ CD8^+^ T cells. In addition, TCF‐1 was also linked to Nur77 expression in human CD8^+^ T cell subsets. These differences between early‐ versus late‐differentiated cells might reflect a heightened sensitivity to TCR stimulation in the former population. Moreover, this divergence may be related to differences in the transcriptional program in response to antigen priming versus restimulation, where negative feedback mechanisms may be induced more robustly in first‐primed early‐differentiated responses to limit immunopathology [23].

Previous studies have shown that Nur77 is involved in regulating T cell tolerance and exhaustion in contexts of chronic antigen stimulation [11]. For instance, Nur77 deficiency in mice enhances antitumor immunity by reducing T cell tolerance and exhaustion markers like PD‐1 and TIM‐3 while promoting terminal effector differentiation [6, 10]. These results are in line with mouse models of autoimmunity, where Nur77 acts as a molecular brake to prevent excessive proliferation and cytokine production, thus conferring protection [4]. In addition, Nur77 deletion in mice promotes the development of systemic inflammation [24]. In humans, Nur77 expression also marks self‐reactive arthritogenic T cells exposed to chronic antigen stimulation [25]. Together, these findings indicate that Nur77 acts as a transcriptional regulator of T cell metabolism and effector differentiation, preventing exacerbated immune responses in context of chronic antigen stimulation, but also impairing effector antitumor responses. On the other hand, the regulatory function of Nur77 could be crucial in maintaining long‐term effective T cell responses. For instance, CD62L^hi^ TCF‐1^hi^ stem‐like memory CD8^+^ T cells in mice actively regulate their anabolic metabolism to prevent cellular damage, and Nur77 may be implicated in this process [12]. Moreover, NR4A1 deficiency in mice impairs the generation and maintenance of Tpex in the tumor environment, augmenting the proportion of Tex cells [13]. Thus, although NR4A1‐deficient cells may exhibit accelerated proliferation and cytotoxicity early upon contact with target cells, their lower survival may limit their long‐term efficacy.

Our study provides novel insights into the role of Nur77 expression in human CD8^+^ T cells in the context of acute, acute resolved, chronic viral infection, and vaccination. In keeping with the notion that Nur77 could be crucial in maintaining long‐term memory CD8^+^ T cell responses, we demonstrate that functionally competent virus‐specific memory CD8^+^ T cells also express Nur77 upon antigen restimulation. Cells expressing Nur77 exhibited higher expression of CCR7 and TCF‐1, better proliferative capacity, and cytokine polyfunctionality compared to their Nur77^−^ counterparts. These phenotypic and functional properties, previously associated with stem‐like memory CD8^+^ T cells [26], were consistently observed in SARS‐CoV‐2‐, vaccine HBV‐, HIV‐, and chronic HBV‐specific CD8^+^ T cells, suggesting a generalizable association between Nur77 and memory‐like virus‐specific T cells. The lower levels of CD127 (also linked to memory CD8^+^ T cells) and higher T‐bet observed in Nur77^+^ cells may be related to the ability of these cells to transition to effector‐like states upon antigen restimulation [27, 28]. Of note, robust polyfunctional stem‐like memory CD8^+^ T cell responses have been associated with asymptomatic or mild COVID‐19 [29], with the protective effect of HBV vaccines [30], and with natural resistance against HIV and HBV chronic infections [31, 32]. In contrast, during acute DENV infection, where the virus‐specific CD8^+^ T cell response is enriched in effector populations, Nur77 expression did not distinguish subsets of virus‐specific CD8^+^ T cells in terms of functional capabilities or phenotypic markers. Moreover, we readily observed a positive correlation between the frequency of Nur77^+^ cells and the magnitude of the central memory response among virus‐specific CD8^+^ T cells. Collectively, our observations suggest that Nur77 expression per se does not necessarily define CD8^+^ T cell dysfunction or exhaustion, particularly in contexts of chronic antigen stimulation. Rather, Nur77 expression appears to define a functional spectrum of antigen‐specific human memory CD8^+^ T cells.

Understanding how Nur77 transcriptional networks influence T cell differentiation and function may provide insights into potential therapeutic targets for modulating immune responses in various disease settings. Here, we observed that the Nur77 modulator Csn‐B induced a TCF‐1^+^ phenotype in human CD8^+^ T cells, as well as potentiated the effect of cell reprogramming with a GSK3 inhibitor. These results are in keeping with recent studies showing that Nur77 activates the Wnt/β‐catenin signaling pathway (of which TCF‐1 is part) in bone marrow mesenchymal stem cells [33]. On the other hand, NR4A family members are repressed by GSK3 [34], which is consistent with the upregulation of Nur77 observed in human CD8^+^ T cells reprogrammed with a GSK3 inhibitor. Thus, Nur77 and related transcriptional circuits seem to play an important role in the differentiation and/or maintenance of memory‐like TCF‐1^+^ human CD8^+^ T cells. Notably, Csn‐B promoted a Nur77^+^ TCF‐1^+^ population with increased polyfunctionality, and this effect was particularly observed in early‐differentiated human CD8^+^ T cells. In addition, Csn‐B enhanced the overall frequency and cytokine polyfunctionality of dysfunctional HIV‐ and HBV‐specific CD8^+^ T cells. This effect may be mediated at least in part by the increased survival and proliferative capacity induced by Csn‐B. Thus, we propose that Csn‐B may be of potential interest for interventions aimed at promoting polyfunctional memory‐like human CD8^+^ T cell responses, such as viral infections and vaccination. Moreover, Csn‐B might improve the therapeutic efficacy of adoptive therapies. Indeed, memory‐like responses have been associated with increased efficacy in CAR‐T cell therapies [35, 36]. Future studies should investigate the molecular mechanisms by which Nur77 exerts these effects in memory‐like CD8^+^ T cells in humans, including the potential Nur77‐TCF‐1 interaction and cell metabolism. Additionally, further research is needed to clarify other signaling pathways that may be affected by Nur77 modulators like Csn‐B.

The following are the limitations of our study: (a) As all analyses were performed ex vivo, further study is needed to elucidate the in vivo therapeutic potential and lifespan of human CD8^+^ T cells under Nur77 modulation; (b) considering that nonactivated cells do not express Nur77, nearly all of this work was performed in CD8^+^ T cell populations after 12 h to 6 days of culture and restimulation. Moreover, we performed 6‐day stimulation assays to simultaneously assess cytokine production and proliferation, a timepoint where Nur77 expression is relatively low compared to 6 h. Thus, our day‑6 antigen‑specific readouts capture residual or re‑induced Nur77 expression at the time of analysis, rather than allowing us to directly track those cells that were highest expressors at the early peak. In addition, as phenotype could change during in vitro stimulation, it is unclear if the Nur77 relationship with a memory‐like phenotype is readily observed in vivo or ex vivo; (c) variations in TCR stimulation strength among individual CD8^+^ T cells may partly account for the observed association between Nur77 expression and cytokine production. Under our in vitro stimulation conditions, cells experiencing stronger TCR engagement are likely to upregulate Nur77 more substantially and exhibit enhanced functional responses. Thus, although Nur77 expression correlates with increased polyfunctionality, this relationship may, at least in part, reflect heterogeneity in stimulation strength; (d) given that previous studies have demonstrated increased expression of Nur77 in mouse tumor‐infiltrating T cells, it might be possible that forced or increased expression of Nur77 in tissues might mark less functional CD8^+^ T cells. Thus, future studies should also include tissue data; (e) we only evaluated the impact of Csn‐B treatment in CD8^+^ T cells from individuals with HIV/HBV coinfection who were on antiretroviral therapy and had undetectable viral loads, a context in which virus‐specific T cells are known to undergo at least partial functional reinvigoration, particularly when therapy is initiated in acute infection [37]. Consequently, Csn‐B–mediated enhancement of HIV‐ and HBV‐specific CD8^+^ T cell function may be limited in untreated individuals with more pronounced residual dysfunction. Although people on antiretroviral therapy would be the main target population for these immunotherapies given the well‐known benefits of treatment initiation, extrapolation of our findings to more dysfunctional T cells in untreated chronic infection should be made with caution and will require dedicated functional studies in viremic cohorts. (f) We cannot exclude potential off‐target or indirect effects of Csn‐B. For instance, prior studies showed that Csn‐B inhibits signal transducer and activator of transcription 3 (STAT3) phosphorylation, impairs Th17 differentiation, and alleviates experimental arthritis in mice [38]. As STAT3 promotes CD8^+^ T cell effector differentiation [39], it is conceivable that Csn‐B favors the generation of memory‐like CD8^+^ T cells through indirect inhibition of STAT3 phosphorylation.

In conclusion, our study indicates that Nur77 is associated with memory‐like and polyfunctional properties in human CD8^+^ T cells. Nur77 represents a potential druggable target, although further validation is required to confirm its suitability for therapeutic modulation. This work enhances our understanding of factors regulating human memory CD8^+^ T cells and may help guide future strategies to improve CD8^+^ T cell functionality in the settings of viral infections, vaccinations, or adoptive therapies.

## 4 Materials and Methods

### 4.1 Study Approval

This study was reviewed and approved by the ethics committee of the School of Medicine, Pontificia Universidad Javeriana, the ethics committee (Comité de Protection des Personnes) of Île‐de‐France XI, and the ethics committee of Clínica Medilaser, Neiva. All study participants provided informed written consent. In addition, in the case of the children included, formal written consent was obtained from the parent/guardians.

### 4.2 Participants

Here, we included buffy coats from healthy donors obtained from two blood banks (Banco Distrital de Sangre, Bogotá, Colombia, and the Établissement Français du Sang, Paris, France). Routine screening tests for HIV‐1/2, hepatitis C, hepatitis B, Chagas disease, syphilis, and HTLV were performed to ensure that blood donors did not have any acute or chronic infection. For the evaluation of antigen‐specific CD8^+^ T cells, three groups of individuals were included: (a) adults with a history of SARS‐CoV‐2 infection and without immunization with an inactivated SARS‐CoV‐2 vaccine; these individuals also had immunization against HBV, using a recombinant hepatitis B surface antigen (HBsAg)‐based vaccine; (b) children with acute DENV natural infection; and (c) adults with chronic HIV and HBV coinfection.

### 4.3 Samples

Individuals with a history of SARS‐CoV‐2 infections were recruited on the basis of previous antigen or nucleic acid amplification tests (NAAT) positive for this virus. These individuals also had a previous complete scheme of recombinant HBV vaccine (Table S1). In the case of children with confirmed dengue, samples were obtained between the third and seventh day after fever onset; their clinical characteristics are shown in Table S2. Participants with confirmed chronic HIV/HBV coinfection were under antiretroviral therapy and with undetectable viral loads (Table S3). No adult donor showed any signs of acute infection at the time of sample collection. A peripheral blood sample was obtained from all the individuals and collected in EDTA‐containing tubes. After blood collection, the cellular fraction was separated by centrifugation at 10,000 rpm for 10 min. Next, PBMCs were isolated by a Ficoll density gradient. Isolated PBMCs were cryopreserved in cell recovery media containing 90% fetal bovine serum (FBS) and 10% DMSO (Sigma‐Aldrich) and stored in liquid nitrogen. Analyses were conducted on cryopreserved PBMCs.

### 4.4 Polyclonal and Antigen‐Specific Stimulation of CD8^+^ T Cells

Frozen PBMCs were thawed and rested overnight at 37°C, 5% CO_2_ in RPMI 1640 supplemented with 20% FBS, penicillin/streptomycin, and glutamax (complete medium). Total PBMCs or purified total CD8^+^ T cells (isolated by negative magnetic isolation [STEMCELL Technologies]) were polyclonally stimulated with plate‐bound anti‐CD3 and CD28 antibodies (ranging from 0.2 to 1 µg/mL; clones OKT3 and CD28.2, respectively; Thermo‐Fisher Scientific), in the presence or absence of human recombinant intercellular adhesion molecule 1 (hrICAM‐1; at 50 µg/mL; R&D Systems) and cultured for up to 48 or 96 h at 37°C, 5% CO_2_. For intracellular cytokine assessment upon polyclonal stimulation, brefeldin A (at 10 µg/mL; Sigma‐Aldrich) was added to the cells and incubated during the last 12 h of cell culture. For antigen‐specific stimulation, total PBMCs derived from each respective group of participants were incubated for 6 days at 37°C, 5% CO_2_ in the presence of (a) DENV CD8 peptide megapool set (at 1 µg/mL) consisting of 268 epitopes with a length of 9–10 amino acids, selected to account for 90% of the IFN‐γ response [20, 40]; (b) a peptide megapool derived from the proteome of the SARS‐CoV‐2 Wuhan ancestral strain (at 1 µg/mL), comprising 621 peptides ranging of 9–10 amino acids, excluding the Spike (S) protein sequence [41]; (c) HBsAg antigen‐derived peptide megapool (at 1 µg/mL) for the analysis of the vaccine‐induced response, comprising 79 epitopes ranging from 8 to 11 residues identified from the Immune Epitope Database (EIDB) [42]; (d) peptide megapools from the HIV (187 peptides) [43] and HBV whole proteomes (core, capsid, P, and X; 189 peptides identified from the IEDB) for the analyses in individuals with chronic coinfection. Peptide megapools were formulated as previously described [44]. Cells stimulated with soluble anti‐CD3 and anti‐CD28 (both at 1 µg/mL) were used as a positive control, and DMSO‐treated cells were used as a negative control. For subsequent intracellular cytokine analysis, cells were restimulated with each peptide pool, accordingly, in the presence of brefeldin A (10 µg/mL) for 12 h. CD8^+^ T cell proliferation was evaluated by CFSE dilution (1 µM, Thermo‐Fisher Scientific). In all cases, cells were cultured at a density of 1 × 10^6^ cells/mL. In independent experiments, purified CD8^+^ T cells were reprogrammed into memory‐like cells using a GSK3 inhibitor, as previously described [18], followed by polyclonal stimulation. In addition, in independent experiments, cells were polyclonally or peptide‐stimulated and simultaneously treated with the Nur77 modulator Csn‐B at 1 µM (MedChemExpress) [21].

### 4.5 Flow Cytometry Analysis

After culture, the cells were stained with the LIVE/DEAD Fixable Aqua Dead Cell Stain kit (Thermo‐Fisher Scientific) along with fluorochrome‐labeled antibodies against human CD3 and CD8. For phenotype analysis, cells were additionally stained with antibodies against CD45RA, CCR7, and CD127 for 20 min at 4°C. Then, the BD Transcription Factor Buffer (BD Biosciences) or the FOXP3/Transcription Factor Staining Buffer Set (Invitrogen) was used for cell fixation and permeabilization. Intracellular staining panels for the detection of transcription factors included fluorochrome‐labeled antibodies against human TCF‐1, Nur77, and T‐bet, along with anti‐IFN‐γ, TNF‐α, and IL‐2 antibodies. Cell acquisition was performed on an LSR Fortessa X‐20 flow cytometer (BD Biosciences), or an Aurora spectral flow cytometer (Cytek). At least 30,000 live CD3^+^ CD8^+^ events were acquired. Flow cytometry data were analyzed with FlowJo software (version 10; BD Biosciences). The list of flow cytometry antibodies used in this study is provided in Table S4.

### 4.6 Statistical and Data Analyses

Data were analyzed in GraphPad Prism software, version 10. In all cases, the median and ranges are shown. Nonparametric tests were used. The Wilcoxon test was applied for the comparison of two paired groups. The Friedman or Kruskal–Wallis test was used to compare three or more paired or unpaired groups, respectively, followed by the Dunn test to correct for multiple comparisons. The Spearman test was used for correlation analyses. In all cases, a *p* value < 0.05 was considered significant.

## Author Contributions

Conceptualization: Fabiola Martel and Federico Perdomo‐Celis. Data curation: Fabiola Martel and Federico Perdomo‐Celis. Formal analysis: Fabiola Martel and Federico Perdomo‐Celis. Funding acquisition: Asier Sáez‐Cirión and Federico Perdomo‐Celis. Investigation: Fabiola Martel, Daniel Rincón, Caroline Passaes, Leonardo Arevalo, Marcela Torres, Natalia Ramirez, and Federico Perdomo‐Celis. Methodology: Fabiola Martel and Federico Perdomo‐Celis. Project administration: Federico Perdomo‐Celis. Resources: Carlos F. Narváez, Jessica F. Toro, Otto Sussmann, Jose Mateus, John Sidney, Alessandro Sette, and Asier Sáez‐Cirión. Supervision: Manuel A. Franco and Federico Perdomo‐Celis. Validation: Fabiola Martel and Federico Perdomo‐Celis. Visualization: Fabiola Martel, and Federico Perdomo‐Celis. Writing – original draft preparation: Fabiola Martel and Federico Perdomo‐Celis. Writing – Review and Editing: Fabiola Martel, Daniel Rincón, Caroline Passaes, Leonardo Arevalo, Marcela Torres, Natalia Ramirez, Carlos F. Narváez, Jessica F. Toro, Otto Sussmann, Manuel A. Franco, Jose Mateus, John Sidney, Alessandro Sette, Asier Sáez‐Cirión, and Federico Perdomo‐Celis.

## Conflicts of Interest

F.P.‐C. and M.A.F. are listed as inventors on a patent application by CellRep Corporation and Pontificia Universidad Javeriana (method for modulating Nur77 in CD8^+^ T cells to enhance their therapeutic potential and applications thereof; USSN 63/794,823). F.P.‐C. and M.A.F. are chief scientific and chief compliance officers at CellRep Corporation. F.M., and D.R., are employees at CellRep Corporation. CellRep Corporation may benefit financially from the results presented in this article. J.M. is an employee at Vividion Therapeutics, San Diego, CA, USA. Other authors declare no conflicts of interest.

## Supporting information




**Supporting File**: eji70202‐sup‐0001‐SuppMat.pdf.

## Data Availability

The data that support the findings of this study are available in the figures and Supporting Information of this article.
