# Supplementary material for: Nur77 Is Associated With Polyfunctional Properties in Virus‐Specific Human CD8+ T Cells
Source: Eur J Immunol. 2026 May 12;56:e70202. doi: 10.1002/eji.70202 (PMC13162089; doi:10.1002/eji.70202)
Supplement: Supplementary file 1 — Supporting File: eji70202‐sup‐0001‐SuppMat.pdf. [file EJI-56-e70202-s001.pdf]

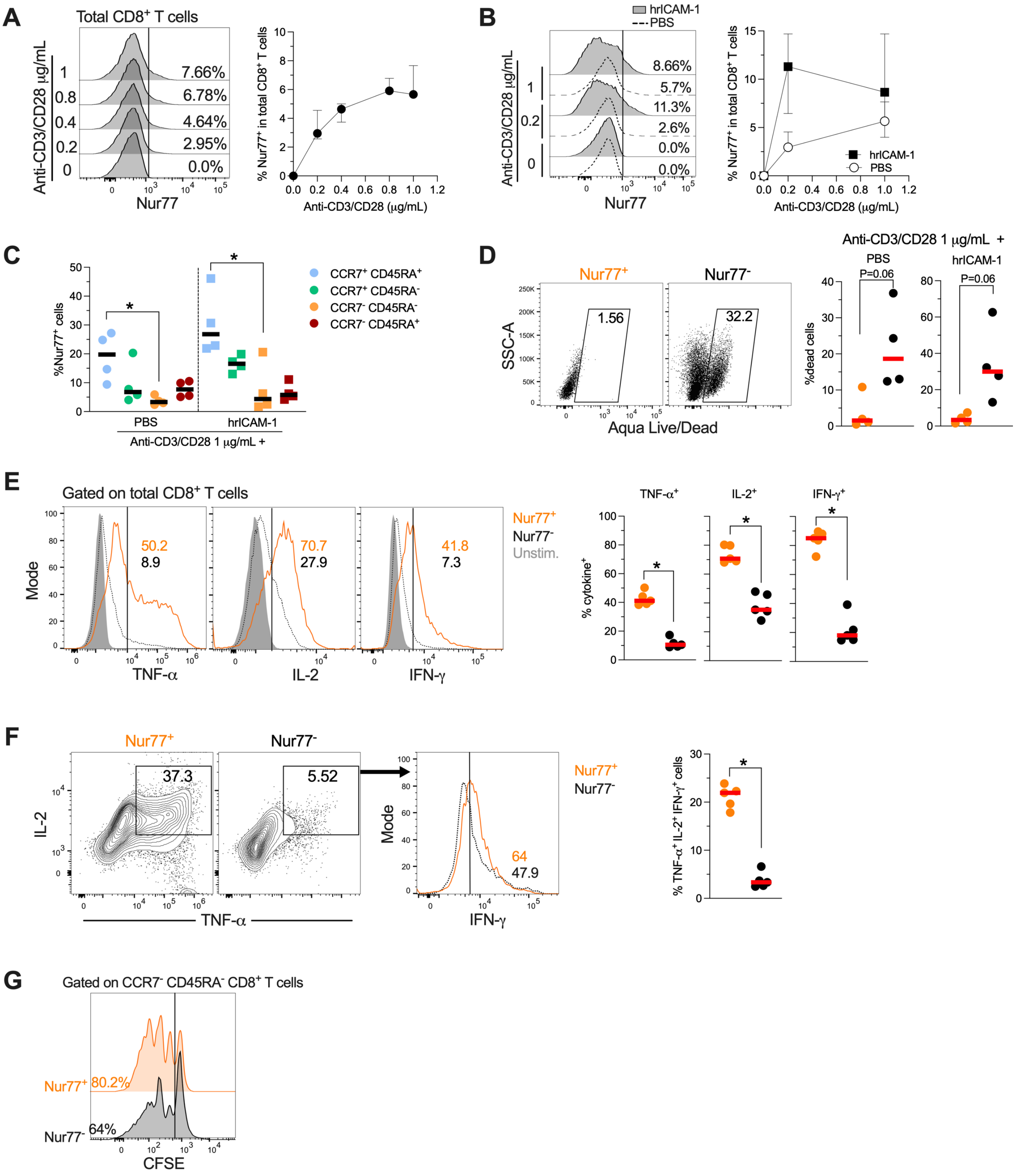

A
Total CD8+ T cells
Anti-CD3/CD28 μg/mL
1
0.8
0.4
0.2
0
7.66%
6.78%
4.64%
2.95%
0.0%
Nur77
% Nur77+ in total CD8+ T cells
Anti-CD3/CD28 (μg/mL)
B
hrICAM-1
PBS
8.66%
5.7%
11.3%
2.6%
0.0%
0.0%
% Nur77+ in total CD8+ T cells
C
%Nur77+ cells
CCR7+ CD45RA+
CCR7+ CD45RA-
CCR7- CD45RA-
CCR7- CD45RA+
PBS
hrICAM-1
Anti-CD3/CD28 1 μg/mL +
D
Nur77+
Nur77-
SSC-A
1.56
32.2
Aqua Live/Dead
Anti-CD3/CD28 1 μg/mL +
PBS
P=0.06
hrICAM-1
P=0.06
%dead cells
E
Gated on total CD8+ T cells
Mode
50.2
8.9
70.7
27.9
41.8
7.3
TNF-α
IL-2
IFN-γ
Nur77+
Nur77-
Unstim.
TNF-α+
IL-2+
IFN-γ+
% cytokine+
F
Nur77+
Nur77-
IL-2
37.3
5.52
TNF-α
Mode
64
47.9
IFN-γ
% TNF-α+ IL-2+ IFN-γ+ cells
G
Gated on CCR7- CD45RA- CD8+ T cells
Nur77+ 80.2%
Nur77- 64%
CFSE

Supplementary Figure 1. Purified CD8$^+$ T cells from healthy donors were stimulated for 48 hs with plate-bound anti-CD3/CD28 antibodies, in the presence or absence of hrICAM-1. **A.** Expression of Nur77 in total CD8$^+$ T cells upon stimulation with increasing concentrations of anti-CD3/CD28 antibodies alone. **B.** Expression of Nur77 in total CD8$^+$ T cells upon stimulation with increasing concentrations of anti-CD3/CD28 antibodies in the presence or absence of hrICAM-1. In A and B, the median and range is shown (n=3). **C.** Expression of Nur77 in CD8$^+$ T cell subsets according to the expression of CCR7 and CD45RA upon stimulation with anti-CD3/CD28 antibodies (1 μg/mL) for 48 hs, in the presence or absence of hrICAM-1. P value of Dunn´s test. **D.** Frequencies of dead cells among Nur77$^+$ and Nur77$^-$ CD8$^+$ T cells, upon polyclonal stimulation for 48 hs. **E.** Frequencies of TNF-α^+, IL-2$^+$, and IFN-γ^+ cells among Nur77$^+$ and Nur77$^-$ total CD8$^+$ T cells, upon polyclonal stimulation for 48 hs. **F**. Frequencies of TNF-α^+ IL-2$^+$ IFN-γ^+ polyfunctional cells among Nur77$^+$ and Nur77$^-$ total CD8$^+$ T cells. **G.** CD8$^+$ T cells were stimulated with anti-CD3/CD28 antibodies (1 μg/mL) for 96 hours. Representative expression of CFSE in Nur77$^+$ and Nur77$^-$ cells. In D-F, P value of Wilcoxon test. Symbols represent one individual and lines indicate the median. Data derived from at least two independent experiments. *P<0.05. NS: Not statistically significant.

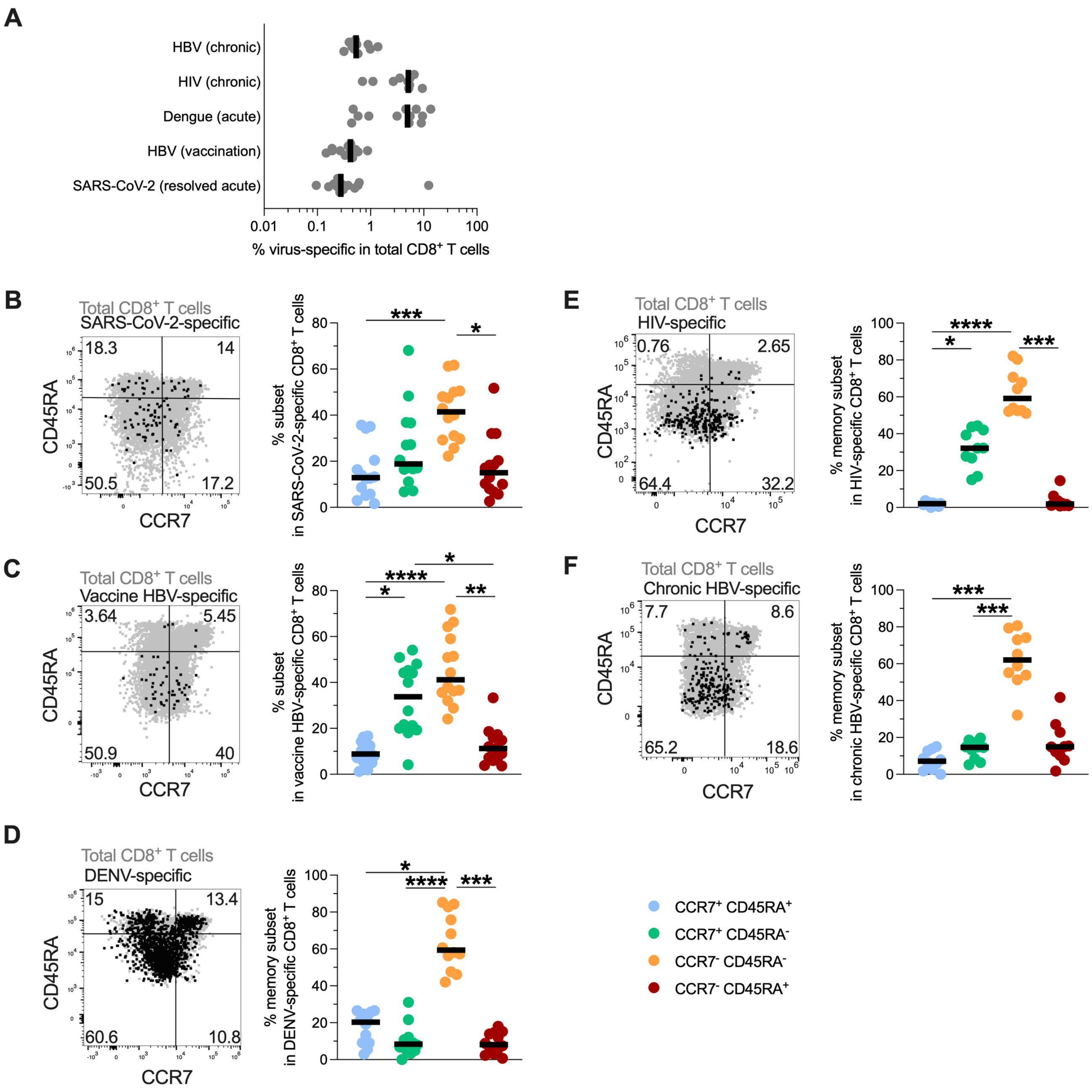
A
HBV (chronic)
HIV (chronic)
Dengue (acute)
HBV (vaccination)
SARS-CoV-2 (resolved acute)
0.01
0.1
1
10
100
% virus-specific in total CD8+ T cells
B
Total CD8+ T cells
SARS-CoV-2-specific
18.3
14
50.5
17.2
CD45RA
CCR7
% subset in SARS-CoV-2-specific CD8+ T cells
***
*
C
Total CD8+ T cells
Vaccine HBV-specific
3.64
5.45
50.9
40
CD45RA
CCR7
% subset in vaccine HBV-specific CD8+ T cells
*
****
*
**
D
Total CD8+ T cells
DENV-specific
15
13.4
60.6
10.8
CD45RA
CCR7
% memory subset in DENV-specific CD8+ T cells
*
****
***
E
Total CD8+ T cells
HIV-specific
0.76
2.65
64.4
32.2
CD45RA
CCR7
% memory subset in HIV-specific CD8+ T cells
****
*
***
F
Total CD8+ T cells
Chronic HBV-specific
7.7
8.6
65.2
18.6
CD45RA
CCR7
% memory subset in chronic HBV-specific CD8+ T cells
***
***
CCR7+ CD45RA+
CCR7+ CD45RA-
CCR7- CD45RA-
CCR7- CD45RA+

Supplementary Figure 2. Peripheral blood mononuclear cells from human donors with previous exposure to SARS-CoV-2 or HBV vaccination, children with acute DENV infection, or people with HIV/HBV chronic coinfection were stimulated for 6 days with a peptide megapool derived from the proteome of the SARS-CoV-2 Wuhan ancestral strain, from the HBsAg, from the DENV proteome, or from HIV or HBV whole proteome, respectively, to assess the corresponding virus-specific $CD8^+$ T cell responses. **A**. Frequency of virus-specific $CD8^+$ T cells. **B-F**. Representative gating strategy and frequencies of memory subsets in SARS-CoV-2- (**B**), vaccine HBV- (**C**), DENV- (**C**), HIV- (**E**), and chronic HBV- (**F**) specific $CD8^+$ T cells. In B-F, P value of Dunn´s test. Symbols represent one individual and lines indicate the median. Data derived from at least two independent experiments. *P<0.05; **P<0.01; ***P<0.001; ****P<0.0001. NS: Not statistically significant.

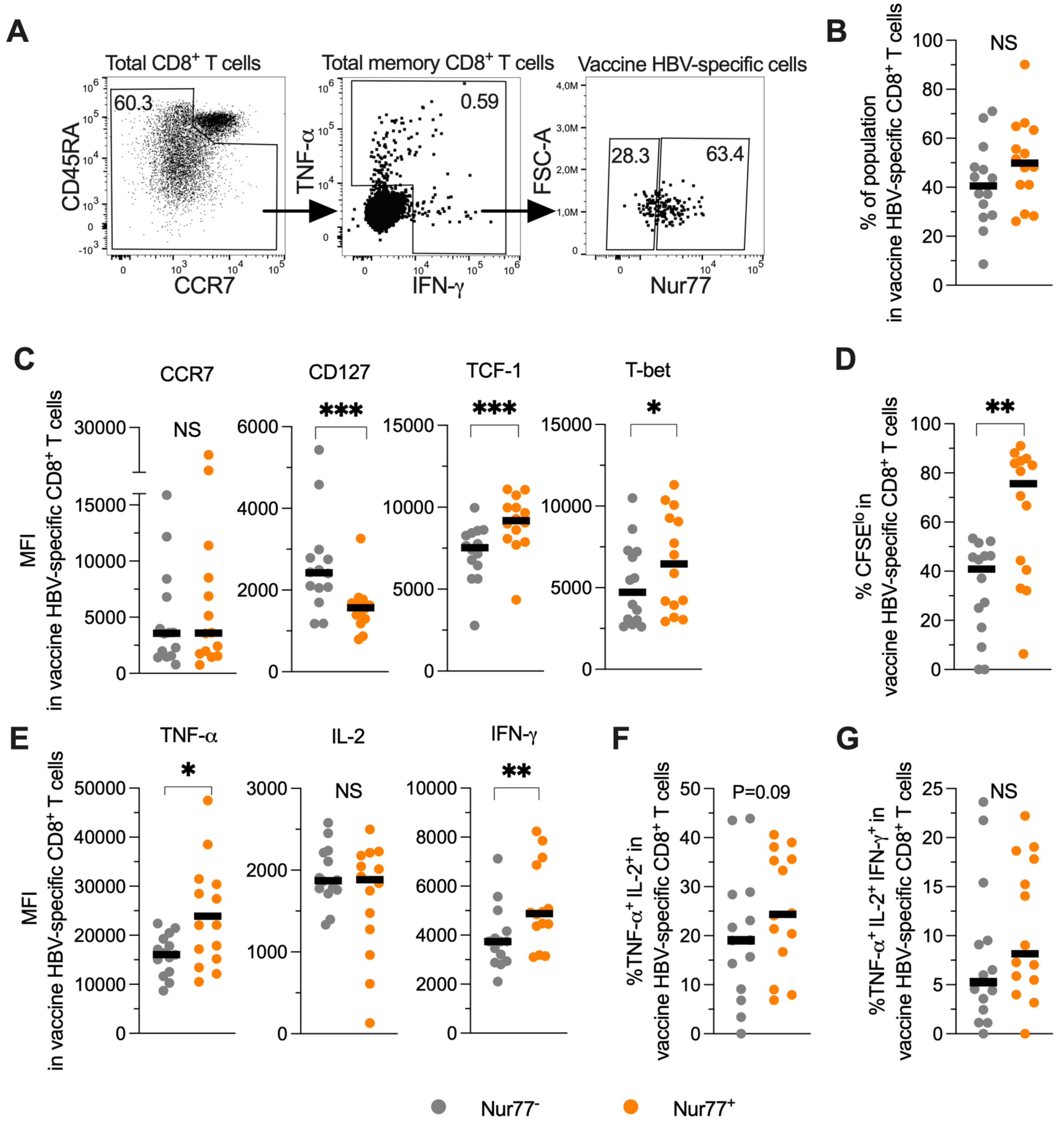
A
Total CD8+ T cells
60.3
CD45RA
CCR7
Total memory CD8+ T cells
0.59
TNF-α
IFN-γ
Vaccine HBV-specific cells
28.3
63.4
FSC-A
Nur77
B
NS
% of population in vaccine HBV-specific CD8+ T cells
C
CCR7
NS
CD127
***
TCF-1
***
T-bet
*
MFI in vaccine HBV-specific CD8+ T cells
D
**
% CFSElo in vaccine HBV-specific CD8+ T cells
E
TNF-α
*
IL-2
NS
IFN-γ
**
MFI in vaccine HBV-specific CD8+ T cells
F
P=0.09
%TNF-α+ IL-2+ in vaccine HBV-specific CD8+ T cells
G
NS
%TNF-α+ IL-2+ IFN-γ+ in vaccine HBV-specific CD8+ T cells
Nur77-
Nur77+

Supplementary Figure 3. Peripheral blood mononuclear cells from human donors with previous HBV vaccination were stimulated for 6 days with a peptide megapool derived from the HBsAg to assess the vaccine-induced $CD8^+$ T cell response. **A.** Representative gating strategy for the analysis of $Nur77^+$ and $Nur77^-$ HBV-specific $CD8^+$ T cells. **B.** Frequencies of $Nur77^+$ and $Nur77^-$ cells among HBV-specific $CD8^+$ T cells. **C**. Expression of CCR7, CD127, TCF-1, and T-bet in $Nur77^+$ and $Nur77^-$ HBV-specific $CD8^+$ T cells. **D.** Frequency of $CFSE^{lo}$ HBV-specific $CD8^+$ T cells. **E**. Expression of TNF-α, IL-2, and IFN-γ in $Nur77^+$ and $Nur77^-$ HBV-specific $CD8^+$ T cells. **F-G**. Frequency of TNF-α^+ IL-2^+ (**F**), and TNF-α^+ IL-2^+ IFN-γ^+ (**G**) cells in $Nur77^+$ and $Nur77^-$ HBV-specific $CD8^+$ T cells. In B-G, the P value of Wilcoxon test is shown. Symbols represent one individual and lines indicate the median. Data derived from three independent experiments. *P<0.05; **P<0.01; ***P<0.001. NS: Not statistically significant.

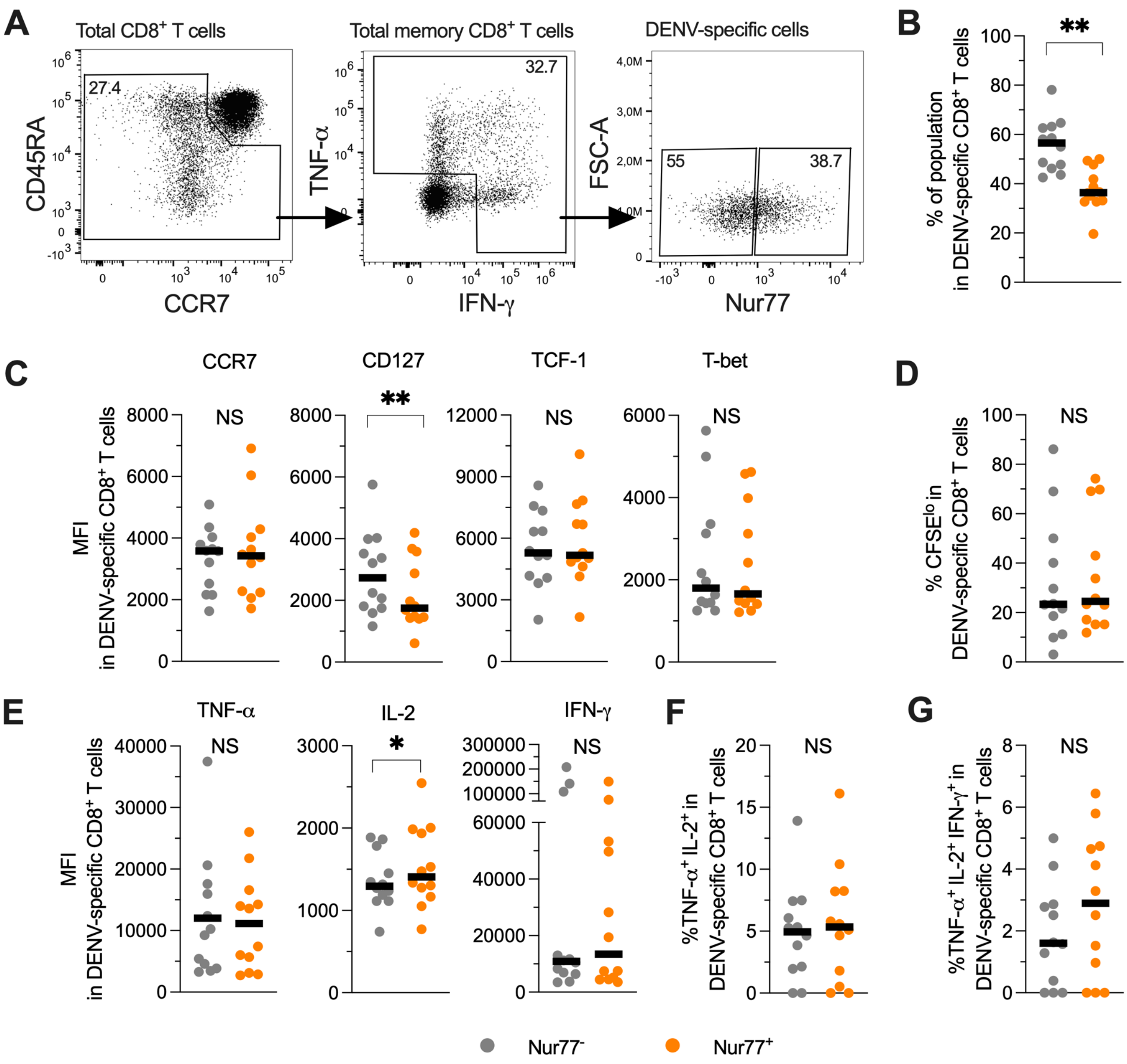

A
Total CD8+ T cells
CD45RA
CCR7
27.4
Total memory CD8+ T cells
TNF-α
IFN-γ
32.7
DENV-specific cells
FSC-A
Nur77
55
38.7
B
% of population in DENV-specific CD8+ T cells
**
C
CCR7
CD127
TCF-1
T-bet
MFI in DENV-specific CD8+ T cells
NS
**
NS
NS
D
% CFSElo in DENV-specific CD8+ T cells
NS
E
TNF-α
IL-2
IFN-γ
MFI in DENV-specific CD8+ T cells
NS
*
NS
F
%TNF-α+ IL-2+ in DENV-specific CD8+ T cells
NS
G
%TNF-α+ IL-2+ IFN-γ+ in DENV-specific CD8+ T cells
NS
Nur77-
Nur77+

Supplementary Figure 4. Peripheral blood mononuclear cells from children with acute DENV infection were stimulated for 6 days with a peptide megapool to assess the corresponding DENV-specific $CD8^+$ T cell responses. **A.** Representative gating strategy for the analysis of $Nur77^+$ and $Nur77^-$ DENV-specific $CD8^+$ T cells. **B.** Frequencies of $Nur77^+$ and $Nur77^-$ cells among DENV-specific $CD8^+$ T cells. **C**. Expression of CCR7, CD127, TCF-1, and T-bet in $Nur77^+$ and $Nur77^-$ DENV-specific $CD8^+$ T cells. **D.** Frequency of $CFSE^{lo}$ DENV-specific $CD8^+$ T cells. **E**. Expression of TNF-α, IL-2, and IFN-γ in $Nur77^+$ and $Nur77^-$ DENV-specific $CD8^+$ T cells. **F-G**. Frequency of TNF-α^+ IL-2^+ (**F**), and TNF-α^+ IL-2^+ IFN-γ^+ (**G**) cells in $Nur77^+$ and $Nur77^-$ DENV-specific $CD8^+$ T cells. In B-G, the P value of Wilcoxon test is shown. Symbols represent one individual and lines indicate the median. Data derived from three independent experiments. *P<0.05; **P<0.01; ***P<0.001. NS: Not statistically significant.

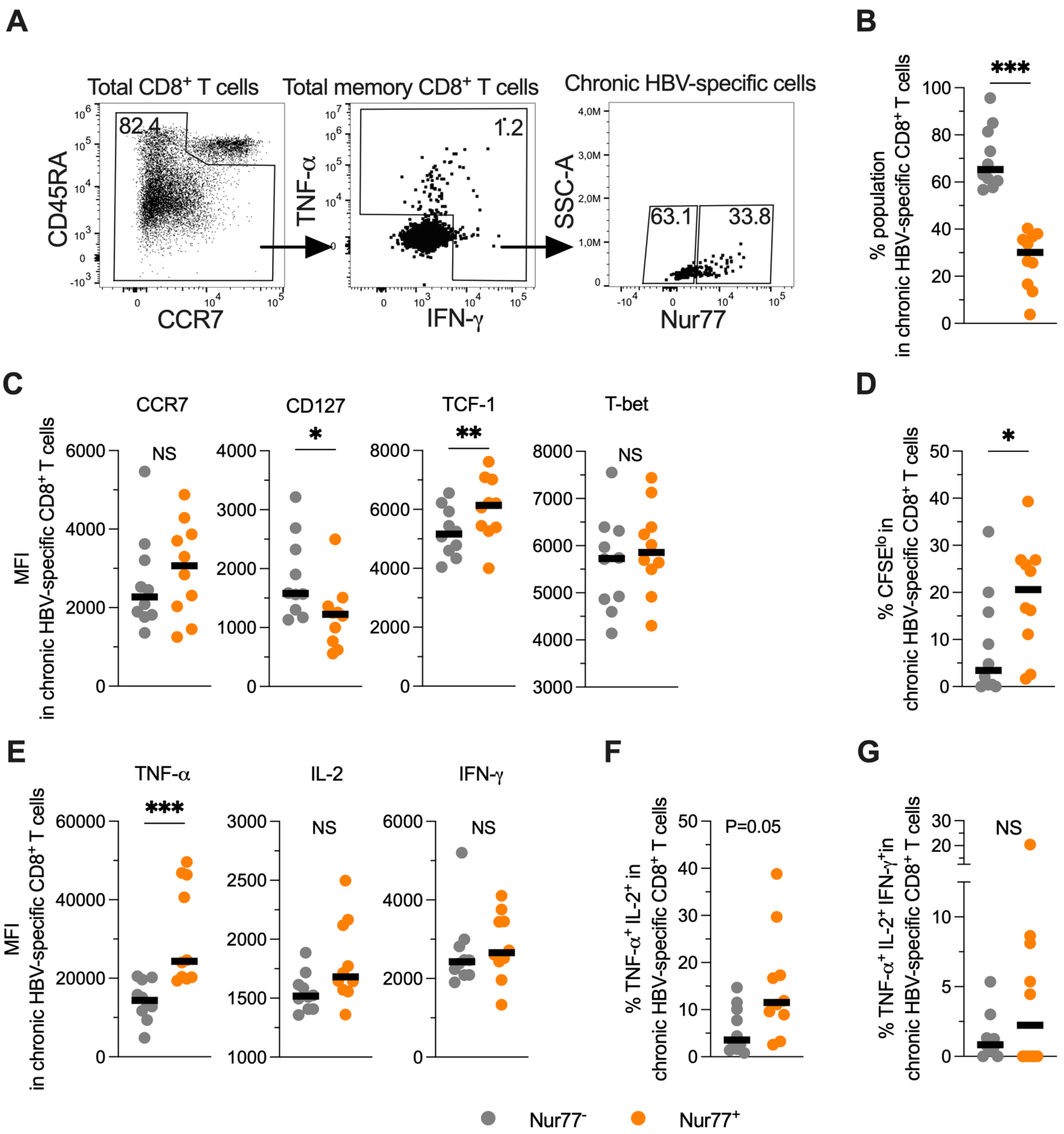
A
Total CD8+ T cells
Total memory CD8+ T cells
Chronic HBV-specific cells
82.4
1.2
63.1
33.8
CD45RA
CCR7
TNF-α
IFN-γ
SSC-A
Nur77
B
% population in chronic HBV-specific CD8+ T cells
***
C
CCR7
CD127
TCF-1
T-bet
NS
*
**
NS
MFI in chronic HBV-specific CD8+ T cells
D
% CFSElo in chronic HBV-specific CD8+ T cells
*
E
TNF-α
IL-2
IFN-γ
***
NS
NS
MFI in chronic HBV-specific CD8+ T cells
F
% TNF-α+ IL-2+ in chronic HBV-specific CD8+ T cells
P=0.05
G
% TNF-α+ IL-2+ IFN-γ+ in chronic HBV-specific CD8+ T cells
NS
Nur77-
Nur77+

Supplementary Figure 5. Peripheral blood mononuclear cells from individuals with chronic HIV/HBV coinfection were stimulated for 6 days with an HBV peptide megapool derived from the whole proteome to assess the corresponding HBV-specific $CD8^+$ T cell responses. **A.** Representative gating strategy for the analysis of $Nur77^+$ and $Nur77^-$ chronic HBV-specific $CD8^+$ T cells. **B.** Frequencies of $Nur77^+$ and $Nur77^-$ cells among chronic HBV-specific $CD8^+$ T cells. **C**. Expression of CCR7, CD127, TCF-1, and T-bet in $Nur77^+$ and $Nur77^-$ chronic HBV-specific $CD8^+$ T cells. **D.** Frequency of $CFSE^{lo}$ chronic HBV-specific $CD8^+$ T cells. **E**. Expression of TNF-α, IL-2, and IFN-γ in $Nur77^+$ and $Nur77^-$ chronic HBV-specific $CD8^+$ T cells. **F-G**. Frequency of TNF-α^+ IL-2^+ (**F**), and TNF-α^+ IL-2^+ IFN-γ^+ (**G**) cells in $Nur77^+$ and $Nur77^-$ chronic HBV-specific $CD8^+$ T cells. In B-G, the P value of Wilcoxon test is shown. Symbols represent one individual and lines indicate the median. Data derived from two independent experiments. *P<0.05; **P<0.01. NS: Not statistically significant.

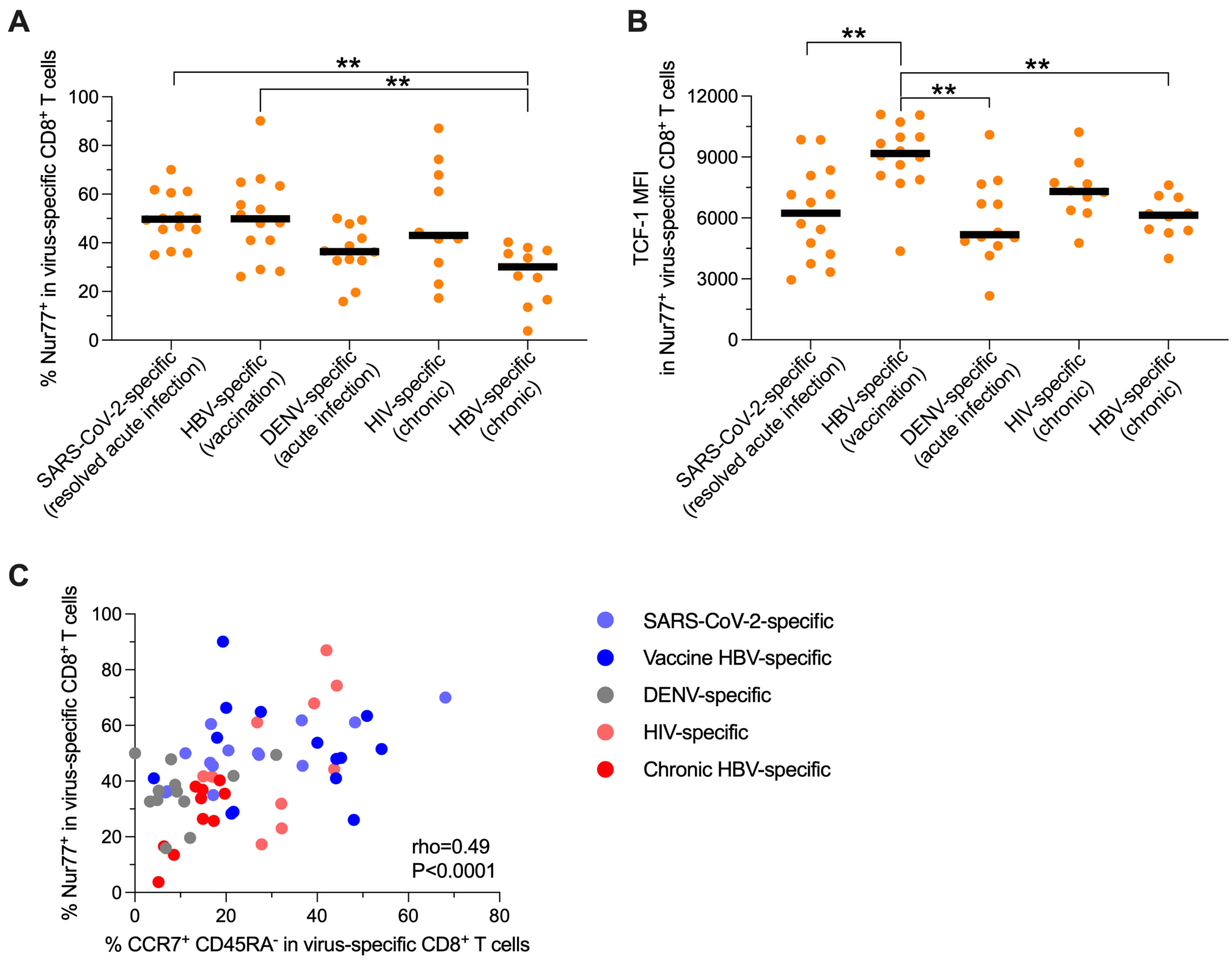

A
B
C
% Nur77+ in virus-specific CD8+ T cells
TCF-1 MFI in Nur77+ virus-specific CD8+ T cells
SARS-CoV-2-specific (resolved acute infection)
HBV-specific (vaccination)
DENV-specific (acute infection)
HIV-specific (chronic)
HBV-specific (chronic)
% CCR7+ CD45RA- in virus-specific CD8+ T cells
SARS-CoV-2-specific
Vaccine HBV-specific
DENV-specific
HIV-specific
Chronic HBV-specific
rho=0.49
P<0.0001

Supplementary Figure 6. A and **B**. Frequency of Nur77$^+$ cells (**A**), and expression levels of TCF-1 (**B**) in virus-specific CD8$^+$ T cells. P value of Dunn´s test. Symbols represent one individual and lines indicate the median. **C.** Correlation analyses between the frequency of Nur77$^+$ cells and the proportion of CCR7$^+$ CD45RA$^-$ among virus-specific CD8$^+$ T cells. Symbols represent one individual; rho and P value of Spearman test. Data derived from at least two independent experiments.

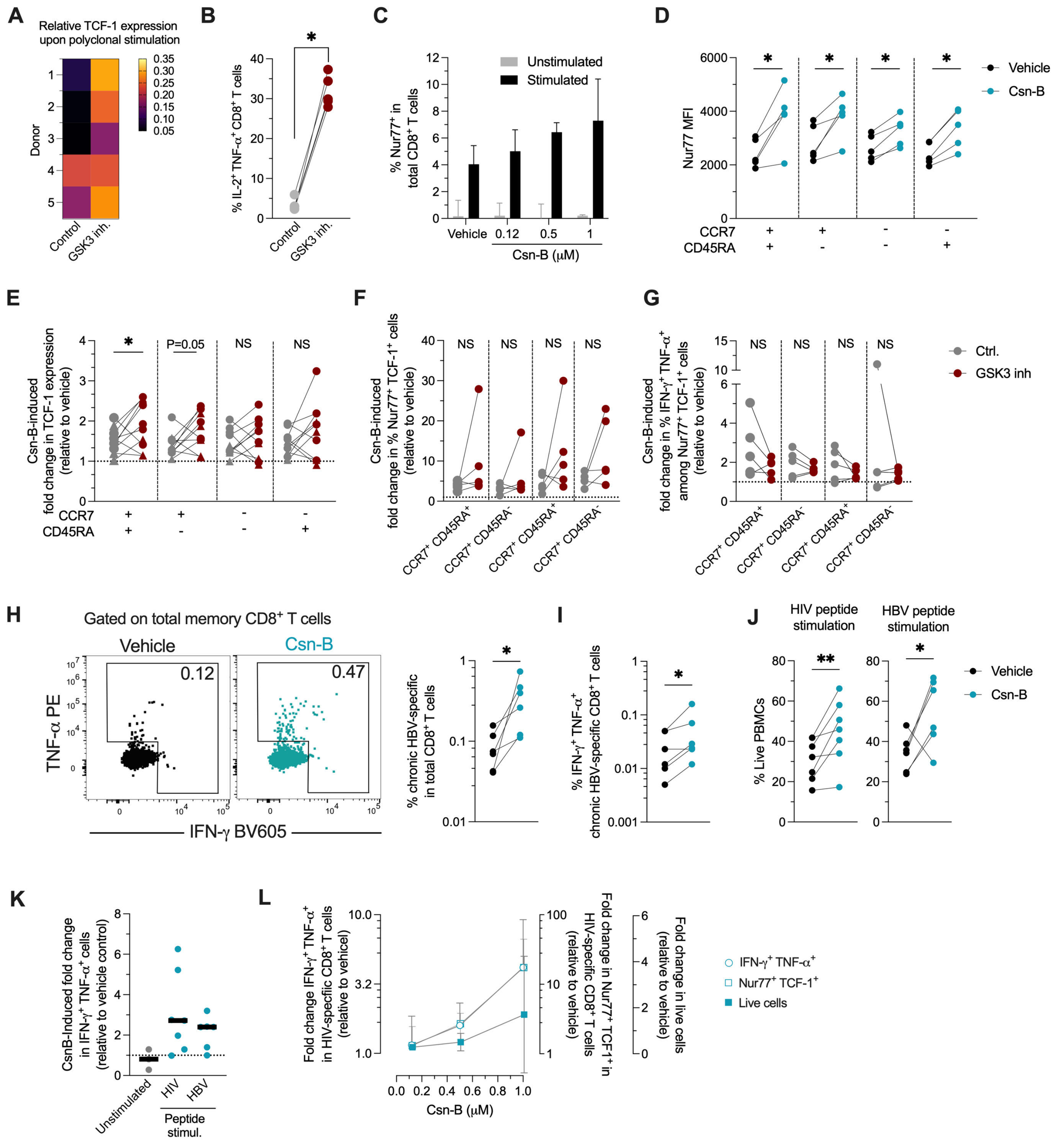
A
Relative TCF-1 expression upon polyclonal stimulation
Donor
1
2
3
4
5
Control
GSK3 inh.
0.35
0.30
0.25
0.20
0.15
0.10
0.05
B
% IL-2+ TNF-α+ CD8+ T cells
Control
GSK3 inh.
C
% Nur77+ in total CD8+ T cells
Unstimulated
Stimulated
Vehicle
0.12
0.5
1
Csn-B (μM)
D
Nur77 MFI
Vehicle
Csn-B
CCR7
CD45RA
E
Csn-B-induced fold change in TCF-1 expression (relative to vehicle)
P=0.05
NS
CCR7
CD45RA
F
Csn-B-induced fold change in % Nur77+ TCF-1+ cells (relative to vehicle)
NS
CCR7+ CD45RA+
CCR7+ CD45RA-
G
Csn-B-induced fold change in % IFN-γ+ TNF-α+ among Nur77+ TCF-1+ cells (relative to vehicle)
Ctrl.
GSK3 inh
H
Gated on total memory CD8+ T cells
Vehicle
Csn-B
0.12
0.47
TNF-α PE
IFN-γ BV605
% chronic HBV-specific in total CD8+ T cells
I
% IFN-γ+ TNF-α+ chronic HBV-specific CD8+ T cells
J
HIV peptide stimulation
HBV peptide stimulation
% Live PBMCs
Vehicle
Csn-B
K
CsnB-Induced fold change in IFN-γ+ TNF-α+ cells (relative to vehicle control)
Unstimulated
HIV
HBV
Peptide stimul.
L
Fold change IFN-γ+ TNF-α+ in HIV-specific CD8+ T cells (relative to vehicel)
Fold change in Nur77+ TCF1+ in HIV-specific CD8+ T cells (relative to vehicle)
Fold change in live cells (relative to vehicle)
Csn-B (μM)
IFN-γ+ TNF-α+
Nur77+ TCF-1+
Live cells

Supplementary Figure 7. A and **B.** Purified $CD8^+$ T cells from healthy donors were treated with vehicle control or reprogrammed with a GSK3 inhibitor, followed by stimulation with anti-CD3/CD28 antibodies and hrICAM-1, for 48 hours. **A**. Expression of TCF-1 upon stimulation. **B**. Frequencies of IL-2^+ TNF-α^+ cells. **C.** Purified $CD8^+$ T cells from healthy donors were polyclonally stimulated in the presence or absence of increasing concentrations of Cytosporone-B (Csn-B), for 12 hours. The expression of Nur77 in total $CD8^+$ T cells is shown. The bar indicates the median and ranges (n=3). **D.** Expression of Nur77 upon polyclonal stimulation in the presence or absence of Csn-B (1 μM). **E-G.** In addition to reprogramming with a GSK3 inhibitor, purified $CD8^+$ T cells from healthy donors were polyclonally stimulated in the presence or absence of Csn-B. The Csn-B-induced fold change in the expression of TCF-1 (**E**), the proportion of $Nur77^+$ TCF-1^+ cells (**F**), and the proportion of polyfunctional $Nur77^+$ TCF-1^+ cells (**G**), relative to non-treated cells, is shown. In E, dots and triangles indicate $Nur77^+$ and $Nur77^-$ cells, respectively. **H** and **I**. Peripheral blood mononuclear cells from people with HIV/HBV coinfection were stimulated for 6 days with an HBV peptide megapool in the presence or absence of Csn-B. **H.** Left: representative expression of IFN-γ and TNF-α for the analysis of HBV-specific $CD8^+$ T cells. Right: frequency of HBV-specific $CD8^+$ T cells in CsnB-treated and untreated cells. **I.** Frequency of IFN-γ^+ TNF-α^+ HBV-specific $CD8^+$ T cells. **J**. Frequency of live PBMCs upon HIV or HBV peptide stimulation of cells derived from people with HIV/HBV coinfection, in the presence or absence of Csn-B. **K**. CsnB-induced fold change in the frequency of IFN-γ^+ TNF-α^+ cells upon HIV or HBV peptide stimulation, or in the absence of peptide stimulation (samples from people with HIV/HBV coinfection were included). **L.** Peripheral blood mononuclear cells from people with HIV/HBV coinfection were stimulated with HIV-derived peptides in the presence or absence of increasing concentrations of Csn-B, for 6 days. The proportion of IFN-γ^+ TNF-α^+ and $Nur77^+$

TCF-1^{+} cells in HIV-specific cells, as well as overall cell survival is shown (n=3). In B, D, and E-J, P value of Wilcoxon test. Symbols represent one individual and lines indicate the median. Data derived from at least two independent experiments. *$P<0.05$; **$P<0.001$. NS: Not statistically significant.

Supplementary Table 1. Demographic characteristics of individuals with a history of SARS-CoV-2 natural infection

**Parameter**	**SARS-CoV-2 natural infection (n=15)**
Gender (Male/Female)	7/8
Age, years; median (range)	29 (22 – 50)
Number of infections; median (range)	2 (1 – 4)
Time since last SARS-CoV-2 infection; median (range)	33 (19 – 54)
**SARS-CoV-2 Diagnostic test**	
PCR	11
Antigen rapid test	4
HBV recombinant vaccine, n (%)	15 (100)

Supplementary Table 2. Clinical and laboratory characteristics of children with dengue.

Parameters<br>(at the day of sample collection)	Children with dengue with warning signs (n=12)
Gender (Male/Female)	6/6
Age, years; median (range)	8.5 (0.8 - 14)
Days of illness, median (range)	5 (3 - 7)
Pleural effusion, yes (%)	7 (58.3%)
Leukocytes, cells x 1000/μL; median (range)	4305 (2390 -6830)
Hemoglobin, g/dL; median (range)	14 (12.60 – 18.80)
Hematocrit, %; median (range)	39.4 (36.5 – 46.20)
Platelets, cells x 1000/μL; median (range)	76,000 (35,000 – 107,000)
Alanine aminotransferase, IU/L; median (range)	161.5 (19.00 - 597)
Aspartate aminotransferase IU/L; median (range)	254 (44 – 1,161)

Supplementary Table 3. Clinical characteristics of individuals with chronic HBV/HIV coinfection

Characteristic	Chronic HBV/HIV coinfection (n=10)
Sex, male, n (%)	10 (100)
Age, years, median (range)	47.3 (40-68)
**Clinical Manifestations**	
**Primary Infections**	
HIV infection, n (%)	10 (100)
Chronic hepatitis B, n (%)	10 (100)
**History of other coinfections**	
Syphilis, n (%)	5 (50)
Hepatitis C Virus, n (%)	2 (20)
HPV / anal condylomatosis / anogenital warts, n (%)	2 (20)
**History of metabolic and liver comorbidities**	
Fatty liver / hepatic alterations, n (%)	2 (20)
Dyslipidemia, hypercholesterolemia, prediabetes, obesity, n (%)	2 (20)
**History of opportunistic Infections**	
Cryptococcosis, n (%)	1 (10)
Candidiasis oropharyngeal, n (%)	1 (10)
Herpes zoster, n (%)	2 (20)
Latent tuberculosis, n (%)	1 (10)
**History of Hematologic / Neurologic / Psychiatric / Other comorbidities**	
Neurologic (seizures, neuropathy, Bell’s palsy, migraine), n (%)	4 (40)
Dermatologic (Seborrheic dermatitis, actinic keratosis), n (%)	2 (20)
Low-grade anal lesions (LSIL / LIE / intraepithelial neoplasia), n (%)	2 (20)
Depression, anxiety, mixed disorders, n (%)	1 (10)
Sleep apnea, n (%)	1 (10)
Thyroid alterations, n (%)	1 (10)
Drug-related renal dysfunction, n (%)	1 (10)
History of gastric cancer, drug-related, n (%)	1 (10)
**Liver and serological profile in chronic Hepatitis B virus infection**	
**HBsAg**	
Low level (1 – 2,000 IU/mL), n (%)	6 (60)
Intermediate (2,001 – 5,000 IU/mL), n (%)	3 (30)
High level (>5,000 IU/mL), n (%)	1 (10)
**Liver Function Tests**	
AST, U/L, median (range)	34.9 (18-114.4)
ALT, U/L, median (range)	36.4 (19.7-82.5)
Platelets count x10^3 /μL, median (range)	261.9 (198-352)
**Fibrosis status**	

Mild (F1), n (%)	1 (10)
Moderate (F2), n (%)	2 (20)
Advanced (F3), n (%)	1 (10)
Not evaluated, n (%)	6 (60)
**Immune-related laboratory data in HIV**	
CD4$^+$ (cells/mL), median (range)	469.1 (199-673)
CD8$^+$ (cells/mL), median (range)	773.7 (425-1418)
CD4:CD8 ratio, median (range)	0.56 (0.24-1.97)
Viral load <20 HIV RNA copies/mL (Log <1.3), n (%)	10 (100)
**Antiviral treatment**	
**NRTIs**	
Emtricitabine, n (%)	10 (100)
Tenofovir disoproxil fumarate, n (%)	6 (60)
Tenofovir alafenamide, n (%)	3 (30)
**NNRTIs**	
Efavirenz, n (%)	1 (10)
Rilpivirine, n (%)	1 (10)
**INIs**	
Dolutegravir, n (%)	3 (30)
**PIs**	
Atazanavir, n (%)	3 (30)
Darunavir, n (%)	2 (20)
**Boosters**	
Ritonavir, n (%)	5 (50)

STI – Sexually Transmitted Infection; **HPV** – Human Papillomavirus; **LSIL** – Low-grade Squamous Intraepithelial Lesion; **LIE** – Low-grade Intraepithelial Lesion; **QoL** – Quality of Life; **TB** – Tuberculosis; HBsAg – Hepatitis B surface Antigen; **AST** – Aspartate Aminotransferase; **ALT** – Alanine Aminotransferase; **NRTIs** – Nucleoside/Nucleotide Reverse Transcriptase Inhibitors; **NNRTIs** – Non-Nucleoside Reverse Transcriptase Inhibitors; **INIs / INSTIs** – Integrase Strand Transfer Inhibitors; **PIs** – Protease Inhibitors.

Supplementary Table 4. Detailed information of antibodies used in this study

Molecule	Fluorochrome	Clone	Manufacturer	Ref. No	Dose
CD3	PerCP	SK7	BD	347344	1:50
CD3	APC efluor 780	UCHT1	Thermo Fisher	47-0038-42	1:50
CD8	Alexa Fluor 700	RPA-T8	BD	561026	1:200
CD8	PE-CF594	RPA-T8	BD	562282	1:100
CD45RA	APC-H7	HI100	BD	560674	1:322
CD45RA	BV421	HI100	BD	562885	1:80
CCR7	PE-Cy7	3D12	BD	557648	1:80
CD127	BV786	HIL-7R-M21	BD	563324	1:322
IFN-γ	BV605	B27	BD	562974	1:100
TNF-α	PE	MAb11	BD	554513	1:800
TNF-α	PerCP Cy.5	MAb11	BD	560679	1:80
IL-2	PE-CF594	5344.111	BD	562384	1:100
IL-2	APC-R700	MQ1-17H12	BD	565136	1:80
TCF-1	BV421	S33-966	BD	566692	1:50
TCF-1	PE	S33-966	BD	564217	1:20
T-bet	BV711	O4-46	BD	563320	1:80
T-bet	V450	O4-46	BD	561312	1:125
Nur77	Alexa Fluor 647	12.14	BD	566735	1:400
CFSE	CFSE	-	Invitrogen	C34554	1:2,500
Live/Dead	Aqua	-	Invitrogen	L34957	1:800
